# Reciprocal influence of soil, phyllosphere, and aphid microbiomes

**DOI:** 10.1186/s40793-023-00515-8

**Published:** 2023-07-21

**Authors:** Adrian Wolfgang, Ayco J. M. Tack, Gabriele Berg, Ahmed Abdelfattah

**Affiliations:** 1 Institute of Environmental Biotechnology, Graz University of Technology, Petersgasse 12, 8010 Graz, Austria; 2 Department of Ecology, Environment and Plant Sciences, Stockholm University, 106 91 Stockholm, Sweden; 3 Present Address: Leibniz Institute for Agricultural Engineering and Bioeconomy (ATB), Max-Eyth-Allee 100, 14469 Potsdam, Germany; 4 Institute for Biochemistry and Biology, University of Potsdam, Karl-Liebknecht-Str. 24-25, 14476 Potsdam, Germany

**Keywords:** Plant–insect-microbe, Multitrophic interaction, Herbivory, Microcosm, *Quercus robur* (pedunculate oak), *Tuberculatus annulatus* (common oak aphid)

## Abstract

**Background:**

The effect of soil on the plant microbiome is well-studied. However, less is known about the impact of the soil microbiome in multitrophic systems. Here we examined the effect of soil on plant and aphid microbiomes, and the reciprocal effect of aphid herbivory on the plant and soil microbiomes. We designed microcosms, which separate below and aboveground compartments, to grow oak seedlings with and without aphid herbivory in soils with three different microbiomes. We used amplicon sequencing and qPCR to characterize the bacterial and fungal communities in soils, phyllospheres, and aphids.

**Results:**

Soil microbiomes significantly affected the microbial communities of phyllospheres and, to a lesser extent, aphid microbiomes, indicating plant-mediated assembly processes from soil to aphids. While aphid herbivory significantly decreased microbial diversity in phyllospheres independent of soil microbiomes, the effect of aphid herbivory on the community composition in soil varied among the three soils.

**Conclusions:**

This study provides experimental evidence for the reciprocal influence of soil, plant, and aphid microbiomes, with the potential for the development of new microbiome-based pest management strategies.

**Supplementary Information:**

The online version contains supplementary material available at 10.1186/s40793-023-00515-8.

## Background

Soil microbiomes influence the microbiome associated with plants, which are known to have major implications for plant resilience, growth, and vigor [1–3]. Plants further interact with various invertebrate animals during their lifespan, for instance, soil-inhabiting, pollinating, or herbivorous arthropods. Insect herbivores often depend on their associated microbiome including microbial symbionts, which may provide pivotal nutrients, or detoxify secondary plant metabolites [4]. Interactions between multicellular organisms like plants and aphids consequently lead to concurrence and interactions of two host-associated microbiomes as well [5, 6]. While it has been documented that soil microbiome influences the phyllosphere [7–9] and aphid microbiomes [7], it is yet unclear whether these effects are direct (via surface-attached contaminants) or plant-mediated (through movement of soil microbes via plant-internal tissues, or through the effect of soil type on plant physiology and subsequently on already present endophytic plant or endosymbiotic aphid communities). Furthermore, it is unclear whether reciprocal effects of aphid herbivory to phyllospheres and soil microbiomes are direct (deposition of aphid-associated microbes, or honeydew dropping to soil affecting soil microbiomes) or plant-mediated (plant responses shaping endophytic communities, or affecting root exudation patterns shaping rhizosphere microbiomes). Investigating only plant-mediated interactions between soil, plant, and aphid microbiomes would indicate to what extent plants themselves are able to modulate and shape soil- and aphid-associated microbiota in their environment. This would have major implications for future pest biocontrol options and our general understanding of plant microbiome assembly under biotic stress.

Phyllosphere microbiome assembly starts during seed germination through microbial inheritance [8, 10–12]. During seed germination, a specific set of microorganisms migrate from the seed to the phyllosphere [10]. Subsequent phyllosphere colonizers are then recruited from the surrounding environment, especially from the soil, through horizontal acquisition [7–9, 13–15], but also dust, air, and water [14, 16]. It is yet unclear whether the observed effect of soil microbiomes on the plant, especially on phyllosphere microbiomes, is due to the direct transmission of microorganisms from the environment, or mediated through the plant. Soil physicochemical properties have a substantial effect on the soil microbiome, which can subsequently influence phyllosphere microbiomes [17, 18]. Therefore, understanding direct and plant-mediated effects on plant microbiomes will reveal to what extent plants use present soil microbial diversity for microbiome assembly, and to what extent plant anatomy and physiology can influence the respective plant microbiome.

The aphid microbiome can be divided into primary endosymbionts such as *Buchnera aphidicola*, secondary symbionts, and transient bacteria [19]. While the presence of primary symbionts is guaranteed by vertical transmission [4, 20], the mechanism by which the remaining members of the aphid microbiome are assembled or maintained is yet not fully known [21]. In the current state of knowledge, factors including aphid species identity, plant host species identity, geographical location, aphid predator frequency, and aphid parasitoid frequency are known to influence the composition of the aphid microbiome [21]. Although soil microbial diversity was shown to influence aphid bacterial communities [7], aphid fungal communities were not investigated using culture-independent methods so far. Furthermore, it is difficult to determine whether in vivo observed effects of soil microbiome on aphid microbiomes are due to plant-mediated mechanisms (plant assembly of soil microbiome and subsequent transmission to aphids) or due to environmental contamination, particularly from the soil. The effect of soil microbes on aphids, with plants connecting below- and above-ground microbiomes, may have important consequences for understanding effects on herbivore performance as well as biocontrol approaches.

Information regarding the effect of aphid infestation on phyllosphere microbiomes is limited, but often attributed to the production and deposition of honeydew to phyllosphere surfaces. Honeydew is known to favor sooty mold species [22] and aphids were found to increase the abundance of culturable epiphytic fungi and bacteria on leaves and shoots of several forest tree species [23–25]. Moreover, aphids are known to deposit associated microbes in and on leaves and induce stress responses in plants [26–30]. Plant stress responses affect phyllosphere microbiomes as well [31]. For instance, woolly beech aphid (*Phyllaphis fagi,* L.) infestation leads to a bacterial community shift in beech (*Fagus sylvatica,* L.)[32]. However, to what extent phyllosphere microbiome response upon aphid herbivory depends on the soil microbiome remains elusive.

Reports on the effect of aphid herbivory on the soil microbiome are relatively scarce and partially contradictory. Some studies have shown that aphid herbivory can change the composition of the microbial communities in the rhizosphere [33, 34], while others reported no observable effects [35]. In nature, soil microbiome shifts upon aphid herbivory may be caused either by the throughfall of honeydew, influencing carbon and nitrogen fluxes [32, 36], or by changes in root exudation patterns, known to shape soil microbiomes [31]. While honeydew throughfall promotes microbial activity in soil [37], the effect of changed root exudates is less clear and probably depends on the biotic or abiotic soil characteristics.

**Fig. 1:**
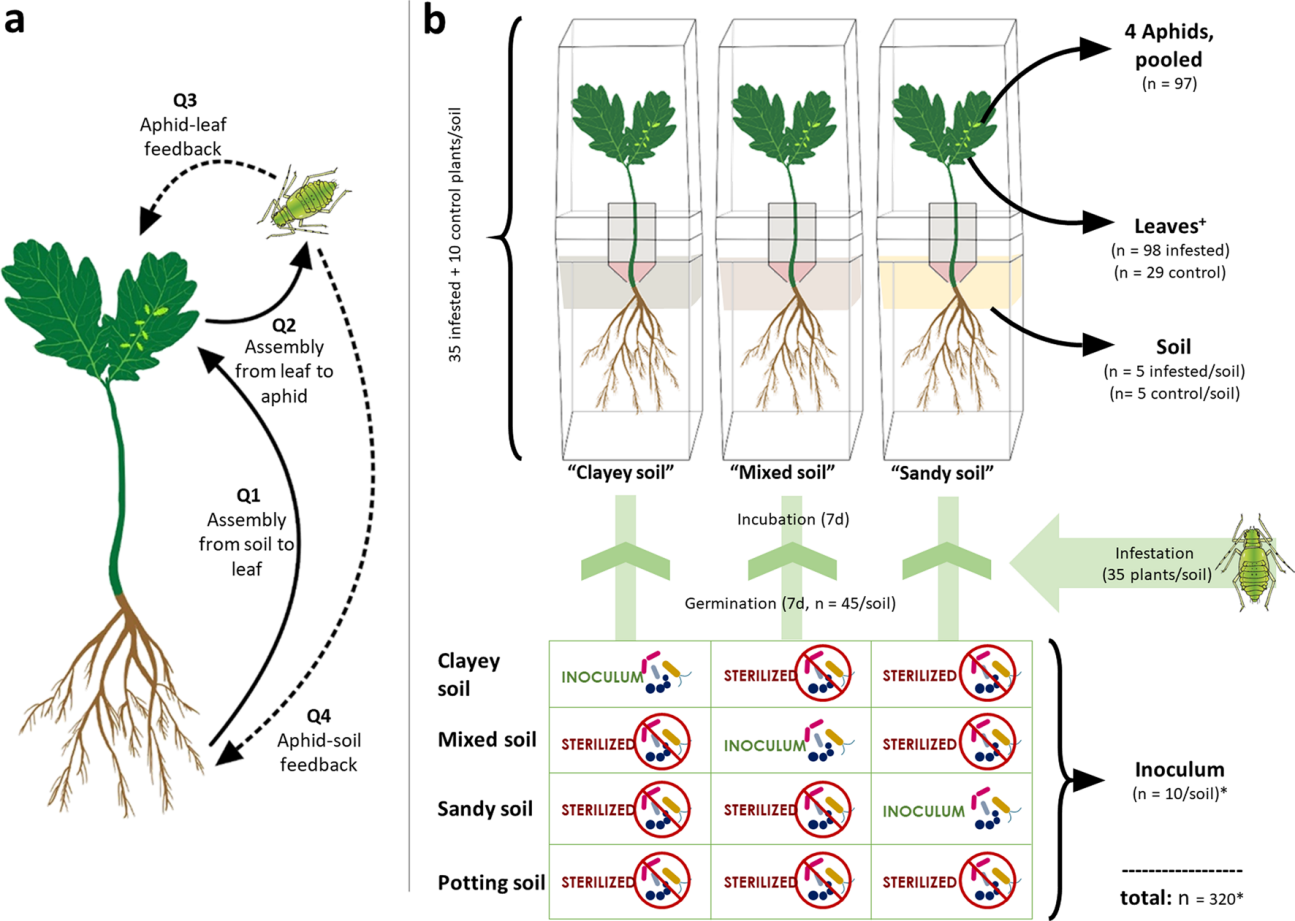
Main research questions (**a**) and experimental setup (**b**). **a** Main research question of the current study. Q1: Do different microbial soil communities lead to differences in phyllosphere communities? Q2: Do different microbial soil communities lead to differences in aphid communities? Q3: Does aphid feeding alter phyllosphere microbial communities? Q4: Does aphid feeding alter soil microbial communities? Assembly processes are depicted as solid arrows, and potential feedback effects are depicted as dashed arrows. **b** Conceptual figure of experimental design, including soil community preparation. The substrate was standardized in physicochemical properties by combining all soils, but with all soils sterilized except for one providing soil community inoculum (bottom). Black arrows indicate DNA extraction for amplicon sequencing of the corresponding microhabitat. ^+^: wilted seedlings and corresponding aphids were removed from the sampling process; therefore “n” refers to the total number of successfully assessed metagenome samples (phyllosphere and aphid samples) or the number of replicate samples per soil in clayey, mixed, and sandy soil microbiomes. *Ten “inoculum” replicates were drawn from microcosms before planting, five replicates per soil type going to be the substrate for aphid-infested plants, and five replicates going to be in the control group

The objective of this study was to examine the effect of the soil microbiome on the assembly of oak phyllosphere and aphid microbiomes, as well as the effects of aphid herbivory on phyllosphere and soil microbiomes. We used the pedunculate oak (*Quercus robur* L.), the common oak aphid (*Tuberculatus annulatus*, HARTIG), and three soil microbial communities established within a physicochemically similar substrate as a test system to answer the following questions:

1. Do different microbial soil communities lead to differences in phyllosphere communities? (Fig. 1a: Q1)
2. Do different microbial soil communities lead to differences in aphid communities? (Fig. 1a: Q2)
3. Does aphid feeding alter the phyllosphere microbial communities? (Fig. 1a: Q3)
4. Does aphid feeding alter soil microbial communities? (Fig. 1a: Q4)

By investigating these questions in a controlled setting, we aim to reveal which formerly observed effects on microbiomes in a plant–herbivore system are truly plant-mediated, and which effects are potentially influenced or influenceable by the soil microbiota in a given soil.

## Materials and methods

### Raw material

Three different types of soil were collected on March 29, 2019, including a loamy sandy soil from the Stockholm University campus (henceforth called’mixed’ soil), a sandy soil, and a clayey soil from Tovetorp Zoological Research Center, situated 60 km southwest of Stockholm. Each soil type was divided into two parts. The first part was autoclaved twice at 120 °C for 20 min with 24-h intervals at room temperature. The second part was used later as inoculum and stored at 4 °C until use (approximately one week). To minimize the effects of soil physicochemical properties, 1.25 l of all three soil types were mixed in the same proportions (v/v) across treatments, with all soil types being sterile except for one soil type, which acted as inoculum for the otherwise identical soil mixtures (Fig. 1b). These mixtures were further separately mixed with 7.5 l of commercial sterilized potting soil (Så och pluggjord, SW Horto, Hammenhög, Sweden) (Additional file 1: Table S1). Since this homogenization method leads to substrates of comparable texture, humidity, macro-, and micronutrient content, we assume the physicochemical background to be the same in the prepared soil mixtures, but differing in colonizing microbial communities [38, 39]. The prepared soil mixtures were kept at 4 °C until use (approximately one week) and are further denoted according to their corresponding non-autoclaved inoculum (‘clayey’,’mixed’, and’sandy’). Two liters of sterilized MilliQ water were added to each soil mix.

Pedunculate oak (*Quercus robur*) acorns were collected from a single oak tree located on the Stockholm University campus (Tree # 000369) to minimize the effect of the plant genotype. Acorns were surface-sterilized to minimize contamination with environment-derived microbes using 5% NaOCl for 30 min, followed by three rinses in sterile MilliQ water each for 10 min. Surface-sterilized acorns were stored in sterile sand at 4 C until use. Before the start of the experiment, acorns were surface sterilized again for 5 min in 5% NaOCl and rinsed as previously mentioned. Common oak aphid (*Tuberculatus annulatus*) was originally collected from natural populations in Stockholm (2018) and reared on oak saplings in a climate chamber (10 h light at 20 °C light, 14 h dark at 18 °C) for several generations prior to the experiment.

### Experimental setup and sample collection

To capture solely plant-mediated microbiome assembly processes, we used microcosms that physically separate above- and below-ground plant compartments to grow seedlings under aseptic conditions [10, 40]. Microcosms included openings with filters in the upper compartments to allow gas exchange, but prevent microbial contaminants from the surrounding **(**Fig. 1b**)**. To separate microbiome shifts in soil due to experimental settings and general plant-mediated effects (e.g. normal root exudation) from herbivory-mediated effects, ten soil samples per soil type, each consisting of 500 mg soil were collected before planting acorns from microcosms going to be in the control group (n = 5) or infested with aphids (n = 5). These samples are further denoted as “inoculum”, despite being the readily prepared soil mixtures at the beginning of the experiment (Fig. 1b). A total of 45 microcosms per soil type were prepared, making a total of 135 microcosms. The lower compartment of the microcosms was filled with 250 ml of soil and left for 10 days in a growth chamber at 20 °C for acclimatization. One surface-sterilized acorn per microcosm was planted under aseptic conditions. Once germinated, a seal was applied to encapsulate the acorn, limiting cross-contamination between below- and above-ground plant parts, preventing neither aphid nor honeydew to come in direct contact with soil, or soil to come in direct contact with neither seedling phyllospheres nor aphids. Seedlings were kept in growth chambers (10 h light at 20 °C, 14 h dark at 18 °C, light intensity 110 µmol m^−2^ s^−1^, air humidity 65%) until they reached the three- to four-leaf stage. For 35 randomly selected seedlings per soil type, twenty aphids were added to the uppermost leaves using a sterile needle. Ten seedlings per soil type were grown without aphids, acting as a control group (Fig. 1b). Microcosms were randomly divided into 4 sampling groups in the course of processing. After seven days, bulk soil, leaves without petioles which were thoroughly checked for aphid remains, and living aphids were collected for DNA extraction. Microcosms containing plants that showed symptoms of wilting or disease were removed from further analyses (Fig. 1b). For bulk soil samples, 500 mg was collected at the center of each microcosm. All samples were stored at − 20 °C until further processing. Leaves were lyophilized using ScanVac CoolSafe™ (LaboGene) and grounded using sterile glass beads and TissueLyser II (Qiagen). Leaf samples are further denoted as “phyllosphere”.

### DNA extraction and library preparation

Inoculum and soil samples were extracted using DNEasy PowerSoil Pro Kit (Qiagen, Hilden, Germany) according to the manufacturer’s instructions. For phyllosphere samples, 200 mg of the lyophilized phyllosphere powder was extracted using DNEasy PowerSoil pro Kit (Qiagen, Hilden, Germany). Aphids were extracted using a modified protocol of the DNeasy® Blood&Tissue (QIAGEN GmbH, Hilden, Germany) standard procedure for insects (Additional file 1: Methods S1). One extraction control sample was added per extraction procedure, which was further treated like additional samples to remove potential contaminants in silico.

Amplification of 16SrRNA and ITS sequences was performed using the primer pairs 515f/806r to amplify a 291 bp bacterial amplicon [41] and ITS1f/ITS2r [42] to amplify fungal amplicons of in average 245 bp. Primers included sample-specific barcodes and Illumina adaptors. For phyllosphere and bulk soil samples, peptide nucleic acid (PNA) PCR clamps were added to block the amplification of plant plastid and mitochondrial DNA [43]. PCR was performed in 30 μl reactions, with a 2 µl template for bulk soil and phyllosphere, and a 5 µl template for aphid samples (Additional file 1: Methods S2). To identify and remove potential contaminants in silico, technical control samples (no-template PCR control samples and extraction control samples for aphid extraction) were also sequenced. In total, 333 and 311 samples for bacteria and fungi were successfully amplified, respectively. PCR products were purified using the Wizard SV Gel and PCR Clean-Up System (Promega, Madison, WI, USA). Final DNA concentrations were estimated using Nanodrop 2000 (Thermo Scientific, Wilmington, DE, USA). Since the source of phyllosphere- and aphid-associated microorganisms is one of the main questions of this study, bulk soil, bacterial phyllosphere, fungal phyllosphere, bacterial aphid, and fungal aphid samples were separately pooled to equimolar concentrations to avoid index hopping [44, 45]. Amplicon sequencing was performed by Eurofins Genomics (Konstanz, Germany) on a MiSeq V3 (600-cycle) platform for 300 bp paired-end sequencing.

### Quantification of fungal and bacterial communities

To quantify the gene copy number of 16S rRNA and ITS rDNA, we used a subset of 4 samples from each treatment for quantitative real-time PCR (qPCR). Target genes were amplified using KAPA SYBR® Green 2X MM (KAPA Biosystems, Cape Town, South Africa) in 10 μl reaction mixtures (for details see Additional file 1: Methods S2). PNA clamps [43] were used for bulk soil and phyllosphere samples. Each measurement was performed in three independent runs on a Rotor-Gene 6000 device (Corbett Research, Mortlake, Australia). Mean fragment copy numbers were blank-corrected and extrapolated to copy numbers per g initial sample weight. We still observed mitochondrial, plastid DNA (16SrRNA dataset), unassigned and plant-assigned reads (ITS dataset) in our amplicon sample results. Therefore, the corresponding relative abundance in the amplicon dataset was used to remove non-target reads from qPCR data. Reads were log10-transformed and will be further denoted as “microbial abundance”.

### Data preprocessing and bioinformatic analyses

Preprocessing of amplicon data was performed in QIIME2 v. 2019.10 [46]. Raw paired-end amplicon sequences were demultiplexed, and primer including adapter were removed using cutadapt [47]. Paired sequences were truncated at 150 bp in bacteria, and at 170 bp fungi respectively, dereplicated, reads merged and denoised using DADA2 [48]. Taxonomy assignment was performed using VSEARCH [49] with SILVA v.132 [50] and UNITE v. 7 [51] as bacterial and fungal reference sequences, respectively. Amplicon sequencing variants (ASVs) table, taxonomy, and metadata were imported to R v. 4.1.1 [52] and further processed using the ‘phyloseq‘ package [53]. For the bacterial dataset, chloroplast, mitochondrial, and reads unassigned at the kingdom level were removed. Due to low remaining ASV read counts in phyllosphere samples, we re-preprocessed the bacterial phyllosphere dataset, using only forward reads with a 200 bp truncation to slightly increase the retained reads after quality filtering. This dataset was used for calculating species richness, Shannon diversity and community composition within the phyllosphere compartment. For comparisons between different compartments (see next section), the complete paired-sequence dataset was used for analyses. For the fungal datasets, plant reads and reads unassigned at the kingdom level were removed. Bacterial and fungal contaminants were identified and removed with the prevalence-based method of the R package’decontam’ using PCR and extraction control samples [54].

### Statistical analyses

Statistical analyses were performed in R v. 4.1.1 [52]. Rarefaction curves were produced using the ‘ranacapa’ package [55]. To account for uneven sequencing depth, ASV tables were rarefied to an even depth of 3900 and 4000 for bulk soil, 100 and 580 for phyllosphere, and 1500 and 4000 for aphid samples for bacteria and fungi, respectively (Additional file 1: Fig. S1). Species richness and Shannon diversity index were estimated using the ‘phyloseq’ package and checked for normal distribution using the Shapiro-Wilks test. For community composition analysis, ASV tables were normalized using Cumulative Sum Scaling using the ‘metagMisc’ package [56] which was used to calculate Bray–Curtis dissimilarities using the ‘phyloseq’ package [53]. Taxonomic assignment correction of missing or uninformative taxa was performed using the ‘miroViz’ package [57].

To test the effect of soils on phyllosphere **(Q1)** and aphid **(Q2)** on microbial community descriptors, we modeled fungal and bacterial richness, Shannon diversity, and abundance as a function of soil type using Kruskal–Wallis test followed by Wilcoxon signed-rank test with FDR-correction for pairwise comparisons. To test the effect of aphid infestation on fungal and bacterial diversity of phyllosphere **(Q3)** and bulk soils **(Q4)**, we modeled fungal and bacterial richness, Shannon diversity, and abundance as a function of aphid infestation using Wilcoxon signed-rank test for all soil types combined and for each soil type separately.

To investigate the effect of soil type on the microbial community composition of phyllosphere **(Q1)** and aphids **(Q2)**, we modeled multivariate fungal and bacterial community composition as a function of soil type, using Bray Curtis distances and the ‘adonis’ function in the vegan package [58]. Pairwise PERMANOVA was performed using the ‘pairwiseAdonis’ package [59] with subsequent Bonferroni correction and was conducted separately for each soil type. To investigate which taxa differed in relative abundance between phyllosphere and aphids grown in different soils, we conducted a Linear discriminant analysis Effect Size (LEfSe) implemented in the’microbial’ package [60, 61].

To investigate the effect of aphid infestation on the microbial community composition of phyllosphere **(Q3)** and bulk soils **(Q4)**, we modeled multivariate fungal and bacterial community composition as a function of aphid infestation, using Bray Curtis distances and the ‘adonis’ function in the ‘vegan’ package [58]. Pairwise PERMANOVA [59] with subsequent Bonferroni correction was conducted separately for each combination of soil type and herbivory. To ascertain that potential differences in microbial community composition in bulk soil due to aphid infestation (**Q4**) do not arise from legacy effects of initial differences in bulk soil communities, we modeled community composition as a function of aphid infestation in the inoculum, comparing bulk soil of control plants and bulk soil of plants being later infested with aphids. To investigate which taxa differed in relative abundance between infested and not infested phyllosphere (**Q3**) and bulk soils (**Q4**), we conducted a Linear discriminant analysis Effect Size (LEfSe) implemented in the’microbial’ package [60, 61]. Potential biomarkers were identified based on Bonferroni-corrected p values (< 0.05), sorted according to LDA score and summarized for each taxonomic rank (phylum, order, family, genus, for fungi only: species). For some LefSe analyses, no biomarker taxa were significantly different after p-value correction; in these cases, we report the most responsive taxa together with p-value significance (corrected or uncorrected) to increase comparability of our results with future research. Using the same method, we identified differential abundant taxa in inoculum and bulk soil to discriminate between general trends in bulk soil community composition due to normal root exudation or experimental settings, and effects mediated by aphid infestation.

To compare the microbial communities between compartments, bacterial and fungal datasets were subset to each combination of compartment and soil type (n = 9), and only samples from aphid-infested plants were kept in the datasets. Each dataset was separately filtered for taxa with at least 50% prevalence using the package ‘metagMisc’ [56], collapsed to genus level and merged into one sample by calculating the mean relative abundance of each genus. Bacterial and fungal datasets were combined and the resulting lists of genera containing their corresponding relative abundances were used to generate a network using Cytoscape v.3.9.1 [62]. The three generated networks contain bulk soil, phyllosphere, and aphids originating from the same soil type. Unique, shared, and partially shared genera were manually arranged for visual clarity and edge width was set according to the relative abundance in the source compartment to visualize the dominance of a given genus in the respective dataset.

## Results

### Amplicon data overview

A total of 6,437,014 bacterial and 13,822,677 fungal reads were retained after quality filtering, decontamination, and removal of plastid DNA, contaminants, and unassigned sequences. A total of 27,596 ASVs were identified in the bacterial amplicon library, and 8671 in fungal amplicon libraries. The maximum read count per sample was 180,761 with a mean of 20,765 reads per sample in bacterial amplicon samples. For fungal amplicon samples, maximum read counts of 542,484 with a mean of 47,338 reads per sample were retained. All three soil inocula differed significantly in community composition both in bacteria (PERMANOVA: R^2^ = 0.43; p = 0.001) and fungi (PERMANOVA: R^2^ = 0.19; p = 0.001). All inocula combined, the bacterial community was dominated by *Proteobacteria*, *Firmicutes*, and *Actinobacteria*; the fungal community was dominated by *Basidiomycota* (dominant genus: *Lyophyllum*) and *Ascomycota* (dominant genus: *Rasamsonia*) (Additional file 1: Notes S1).

### Assembly from soil to phyllosphere

**Fig. 2:**
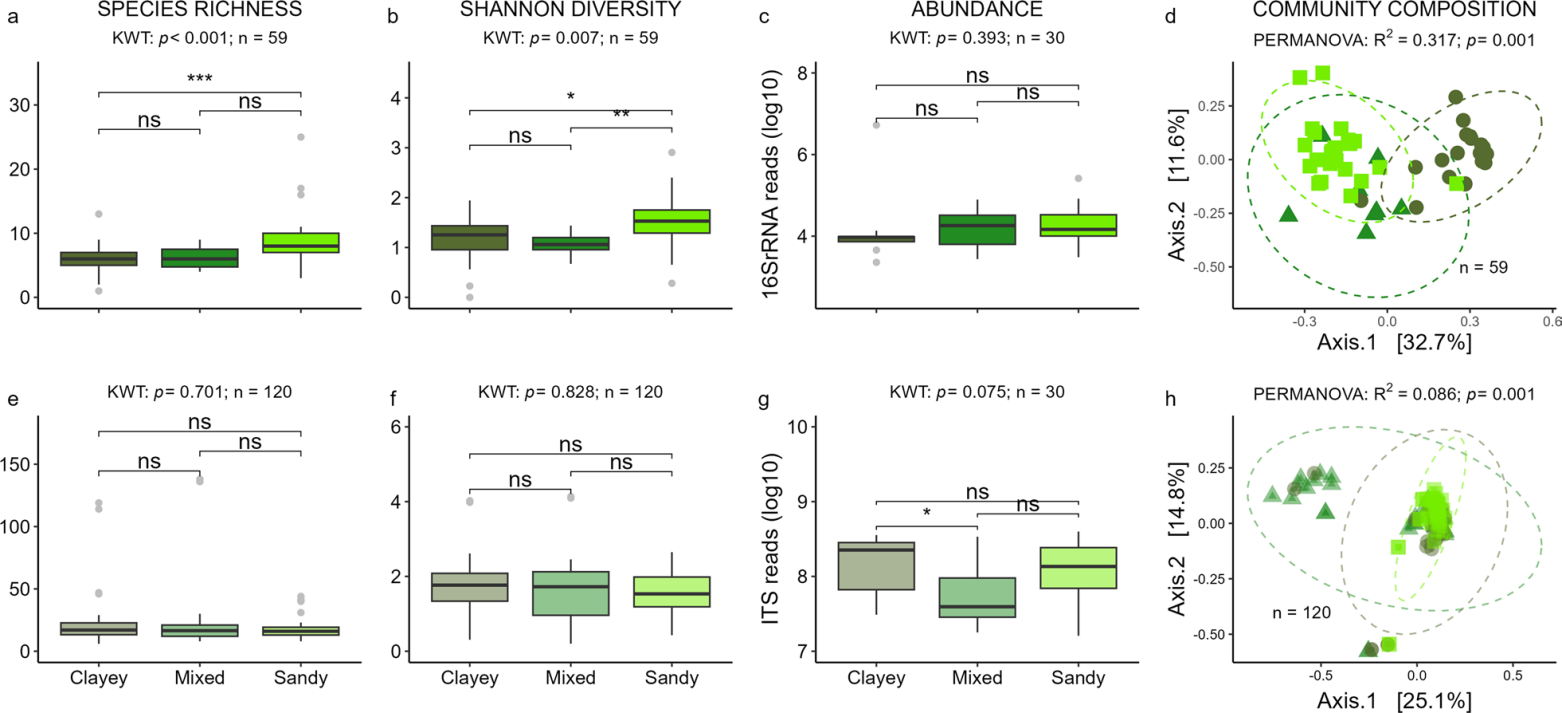
Effect of soil microbiome on phyllosphere microbiomes. Bacterial (top, **a**–**d**) and fungal (bottom, **e**–**h**) species richness (**a**, **e**), Shannon diversity (**b**, **f**), abundance based on qPCR of 16S rRNA (**c**) and ITS read counts (**g**), and community composition (**d**, **h**). Box plots show the median (horizontal line), the lower and upper bounds of each box plot denote the first and third quartiles and whiskers above and below the box plot show 1.5 times the interquartile range. Points located outside of the whiskers (grey) represent outliers. Ordination plots of bacterial (**d**) and fungal **h** community composition based on Bray–Curtis dissimilarity index with corresponding colors (phyllospheres from clayey soil samples: olive circles; mixed: medium green triangles; sandy: lime green squares). X-axes labels of top boxplots **a**–**c** correspond to x-axes labels of lower boxplots (**e**–**g**). Results of global statistical analyses for the factor ‘soil community’ (**a**–**c** and **e**–**g**: Kruskal–Wallis test; **d** and **h**: PERMANOVA) are displayed above each panel, FDR-corrected p-values of pairwise comparisons (Wilcoxon signed-rank test) for alpha diversity differences and abundance **a**–**c**, **e**–**g** added within the graph (*p < 0.05; **p < 0.01; ***p < 0.001; *ns* not significant). *KWT* Kruskal–Wallis test

Soil microbiome had a significanteffect on the bacterial species richness (Fig. 2a) and Shannon diversity (Fig. 2b) in the phyllosphere. Among the different soil types, the phyllosphere of plants grown in sandy soil microbiome displayed the highest bacterial richness (not significant compared to mixed soil) and Shannon diversity. Phyllosphere of plants grown in mixed soil microbiome on average exhibited the lowest bacterial richness (not significant) and Shannon diversity (not significantly different to clayey soil). Bacterial abundance in the phyllosphere did not differ significantly according to soil microbiome (Fig. 2c), but bacterial community composition differed significantly for both overall (PERMANOVA, Fig. 2d) and all pairwise comparisons (pairwise PERMANOVA, Additional file 1: Table S2). Fungal phyllosphere species richness (Fig. 2e) and Shannon diversity (Fig. 2f) were not affected by the soil microbiome, yet fungal abundance was significantly higher in phyllosphere grown in clayey than in mixed soil microbiome **(**Fig. 2g**)**. Fungal phyllosphere community composition significantly differed according to the three soil types (Fig. 2h) microbiomes. Pairwise PERMANOVA revealed fungal phyllosphere communities grown in the three soil microbiomes to significantly differ from each other (Additional file 1: Table S2). Differential abundance analysis showed that *Burkholderia* s. lat. (*Burkholderia-Caballeronia-Paraburkholderia*) is a biomarker for the phyllosphere grown in clayey, *Pseudomonas* for the phyllosphere in mixed, and *Streptomyces*, *Sphingomonas*, *Erwinia,* and *Acinetobacter* for the phyllosphere microbiota in sandy soil microbiomes. High microbial species richness, Shannon diversity, or abundance in soil was rarely co-occurring with high species richness, Shannon diversity, and abundance in the phyllosphere (Table 1).

**Table 1:** Dynamics in alpha diversity during soil microbe assembly. Comparison of bacterial and fungal species richness, Shannon diversity, and abundance between different soil microbiomes relative to each other for the three tested compartments soil, phyllosphere, and aphids. Abundance based on log10 transformed qPCR reads of 16S rRNA (bacteria) and ITS (fungi) gene read counts. Rank position (1 = high, 2 = medium, 3 = low) of richness, Shannon diversity and abundance values compared between corresponding compartments (rows). Compartments where phyllosphere and aphid samples show similar ranks are highlighted in bold

			Bacteria			Fungi		
		Soil microbiome type	Clayey	Mixed	Sandy	Clayey	Mixed	Sandy
Species richness	⇓	Soil (rank)	581.6 (3)	742.8 (1)	644 (2)	33.5 (3)	78.7 (1)	72.9 (2)
		Phyllosphere (rank)	5.83 (3)	6.25 (2)	8.79 (1)	23.26 (1)	22.53 (2)	**18.03 (3)**
		Aphids (rank)	159.78 (2)	158.00 (3)	226.39 (1)	69.92 (2)	78.06 (1)	**45.29 (3)**
Shannon diversity	⇓	Soil (rank)	5.62 (2)	5.95 (1)	5.41 (3)	0.97 (3)	1.77 (1)	1.42 (2)
		Phyllosphere (rank)	1.17 (2)	**1.07 (3)**	1.50 (1)	**1.70 (1)**	**1.63 (2)**	**1.58 (3)**
		Aphids (rank)	1.29 (1)	**1.15 (3)**	1.15 (2)	**2.06 (1)**	**1.80 (2)**	**1.71 (3)**
Abundance	⇓	soil (rank)	8.06 (2)	8.17 (1)	8.05 (3)	5.49 (2)	5.69 (1)	5.18 (3)
		Phyllosphere (rank)	**4.18 (2)**	**4.17 (3)**	**4.30 (1)**	8.15 (1)	7.74 (3)	8.07 (2)
		Aphids (rank)	**4.11 (2)**	**3.87 (3)**	**4.66 (1)**	3.23 (3)	3.52 (2)	4.92 (1)

### Assembly from soil to aphid

**Fig. 3:**
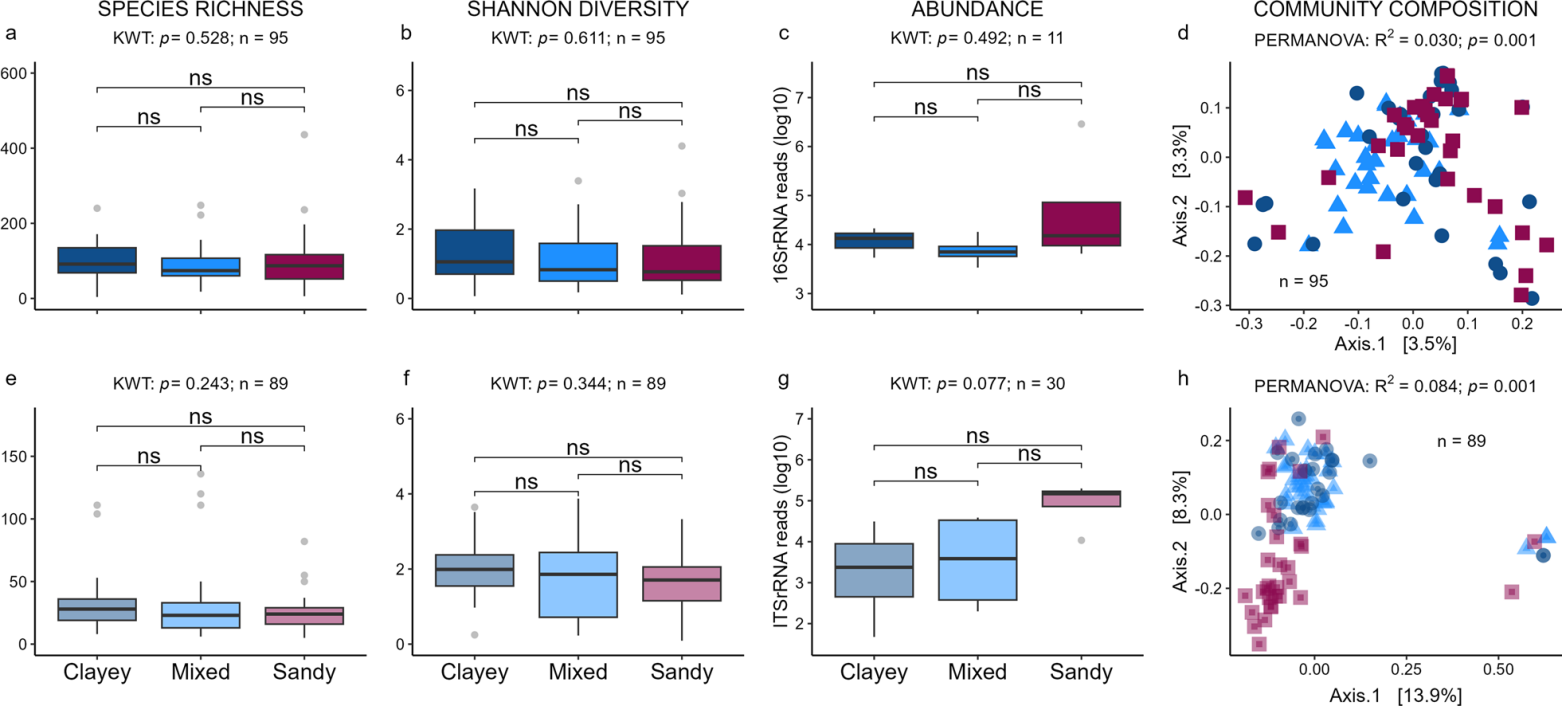
Effect of soil microbiome on pooled (n = 4) aphid microbiomes. Bacterial (top, **a**–**d**) and fungal (bottom, **e**–**h**) species richness (**a**, **e**), Shannon diversity (**b**, **f**), abundance based on qPCR of 16S rRNA (**c**) and ITS read counts (**g**), and community composition (**d**, **h**). Box plots show the median (horizontal line), the lower and upper bounds of each box plot denote the first and third quartiles and whiskers above and below the box plot show 1.5 times the interquartile range. Points located outside of whiskers (grey) represent outliers. Ordination plots of bacterial (**d**) and fungal **h** community composition based on Bray–Curtis dissimilarity index with corresponding colors (aphids from clayey soil samples: dark blue circles; mixed: light blue triangles; sandy: pink squares). Bacterial community composition was calculated with aphid primary endosymbiont *Buchnera aphidicola*. X-axes labels of top boxplots **a**–**c** correspond to x-axes labels of lower boxplots (**e**–**g**). Results of the global statistical analyses (**a**-**c** and **e**–**g**: Kruskal–Wallis test; d and h: PERMANOVA) for the factor ‘soil community’ are displayed above each panel, FDR-corrected p-values of pairwise comparisons (Wilcoxon signed-rank test) for alpha diversity differences and abundance **a**–**c**, **e**–**g** added within the graph (*p < 0.05; **p < 0.01; ***p < 0.001; *ns* not significant). *KWT* Kruskal–Wallis test

Soil did not affect bacterial species richness (Fig. 3a), diversity (Fig. 3b), or abundance (Fig. 3c) in bacterial aphid communities, but significantly affected overall community composition (PERMANOVA, Fig. 3d) in aphids. Pairwise comparisons of bacterial community composition revealed significant differences between all treatments, except for aphids from clayey and sandy soil microbiomes (pairwise PERMANOVA, Additional file 1: Table S3). The same pattern as in bacteria was observed in the fungal aphid microbiome (Fig. 3e–h). The variance explained (R^2^) by the factor soil type in community composition was higher in fungal (Fig. 3h), than in bacterial aphid microbiomes (Fig. 3d). Pairwise PERMANOVA showed a significant difference between soil microbiomes in all fungal communities of aphids (Additional file 1: Table S3). Aphid microbiomes were dominated by *Buchnera, Burkholderia* s. lat., and *Pseudomonas*, while fungal microbiomes were dominated by *Cladosporium* and *Penicillium* (Additional file 1: Notes S2). Differential abundance analyses using LefSE only showed *Micromonosporaceae* to be significantly higher in aphids reared on sandy soil microbiome (Additional file 1: Table S4). In the fungal dataset, *Mortierella* spp. and *Parasola* spp. tend to be more abundant in aphids reared on clayey soil microbiome, while *Cladosporium* spp. was higher in aphids reared on mixed soil microbiome, but p-values were not significant (> 0.05) after Bonferroni correction (Additional file 1: Table S5). High microbial species richness, Shannon diversity, and abundance in bulk soil was rarely co-occurring with high species richness, Shannon diversity, and abundance in aphids, but bacterial abundance and fungal Shannon diversity in aphids showed similar patterns (e.g., species richness is highest in bulk soil type A in both phyllosphere and aphid from the same soil, while it is lowest in bulk soil type B in both aphids and phyllosphere) when compared to bacterial abundance and fungal Shannon diversity in phyllosphere (Table 1).

### The effect of aphid herbivory on phyllosphere microbiota

**Fig. 4:**
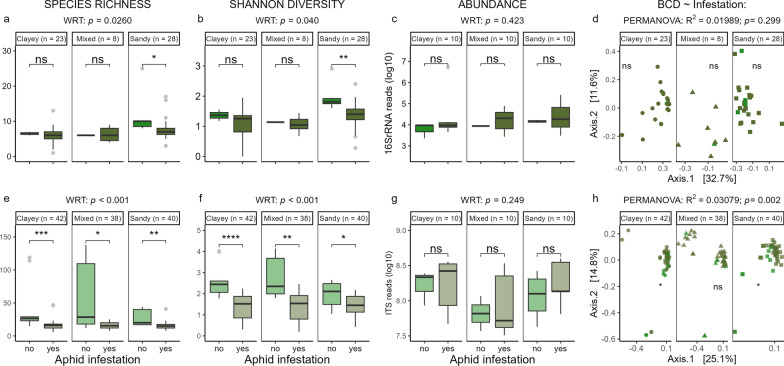
Effect of aphid infestation on phyllosphere-associated microbiomes established in three different soil microbiomes. Bacterial (top, **a–d**) and fungal (bottom, **e**–**h**) species richness (a, d), Shannon diversity (b, e), abundance based on qPCR of 16S rRNA (**c**) and ITS read counts (**g**), and community composition **d**, **h** of phyllosphere communities. Global p refers to differences if all phyllosphere samples were combined. Box plots show the median (horizontal line), the lower and upper bounds of each box plot denote the first and third quartiles and whiskers above and below the box plot show 1.5 times the interquartile range. The points located outside of the whiskers of the box plot (grey) represent outliers. X-axes labels of top boxplots **a**–**c** correspond to x-axes labels of lower boxplots (**e**–**g**). Results of the global statistical analyses for the factor ‘aphid infestation’ (WRT: Wilcoxon signed-rank test) for all phyllosphere samples combined are displayed above each panel, soil community-specific values are displayed within the graph. BCD: Bray–Curtis distance

Aphid herbivory had a significant decreasing effect on the phyllosphere bacterial species richness (Fig. 4a) and Shannon diversity (Fig. 4b). Bacterial abundance (Fig. 4c) and community composition (Fig. 4d) did not differ significantly. In contrast to bacteria, fungal phyllosphere species richness (Fig. 4e) and Shannon diversity (Fig. 4f) was decreased by aphid herbivory, independent of soil microbiome, while fungal abundance was not affected by aphid herbivory (Fig. 4g). Fungal community composition significantly differed between infested and control phyllosphere (Fig. 4h). Pairwise comparisons of fungal community composition showed that aphid herbivory significantly affected fungal phyllosphere communities grown in clayey (R^2^ = 0.045, p = 0.018) and in sandy soil microbiomes (R^2^ = 0.060, p = 0.013), but not in mixed soil microbiomes (R^2^ = 0.045, p = 0.09) (Fig. 4h). LefSe analysis showed that 72 fungal genera were more abundant in the phyllosphere of non-infested compared to infested plants. Among those taxa, *Russula* and *Preussia* were the most affected taxa identified to genus level (*p* value adjusted, Additional file 1: Table S6) and also the entomopathogenic fungus *Metarhizium anisopliae* (LDA: 4.31) was found in the dataset. On the other hand, the phylum *Ascomycota* was significantly more abundant in the aphid-infested phyllosphere but no specific taxon on lower taxonomic ranks was identified (Additional file 1: Table S6).

### The effect of aphid herbivory on bulk soil microbiota

Aphid herbivory did not affect microbial bulk soil species richness, diversity, abundance, and community composition (Additional file 1: Fig. S2), except for bacterial species community composition in sandy soil (Additional file 1: Fig. S2d). According to LefSe analyses results, the effect of aphid infestation on the relative abundance of sandy bulk soil microbiota was not significant after p-value correction for any taxon. The strongest negative response on aphid herbivory was observed for *Rhodanobacter* and -amongst others- *Bacillaceae*. The relative abundance of *Rhizobiaceae* (*Rhizobium* s. lat., *Mesorhizobium*) and *Xanthobacteraceae* was higher in sandy bulk soil of aphid-infested plants, yet all these results were not significant after p value correction (Additional file 1: Table S7). Four bacterial genera significantly increasing from inoculum to bulk soil (*Arenimonas, Ferruginibacter, Terrimonas, Devosia*) were higher in bulk soil from aphid-infested plants than in the control group, yet not significant after p value correction (Additional file 1: Table S8), although a soil type-dependent community development was observed when comparing inoculum and bulk soil microbiomes (Additional file 1: Fig. S3-S5). No specific fungal taxa showed significantly different relative abundances in bulk soil microbiomes of the aphid-infested plant.

### Taxa shared between soil, phyllosphere, and aphids

**Fig. 5:**
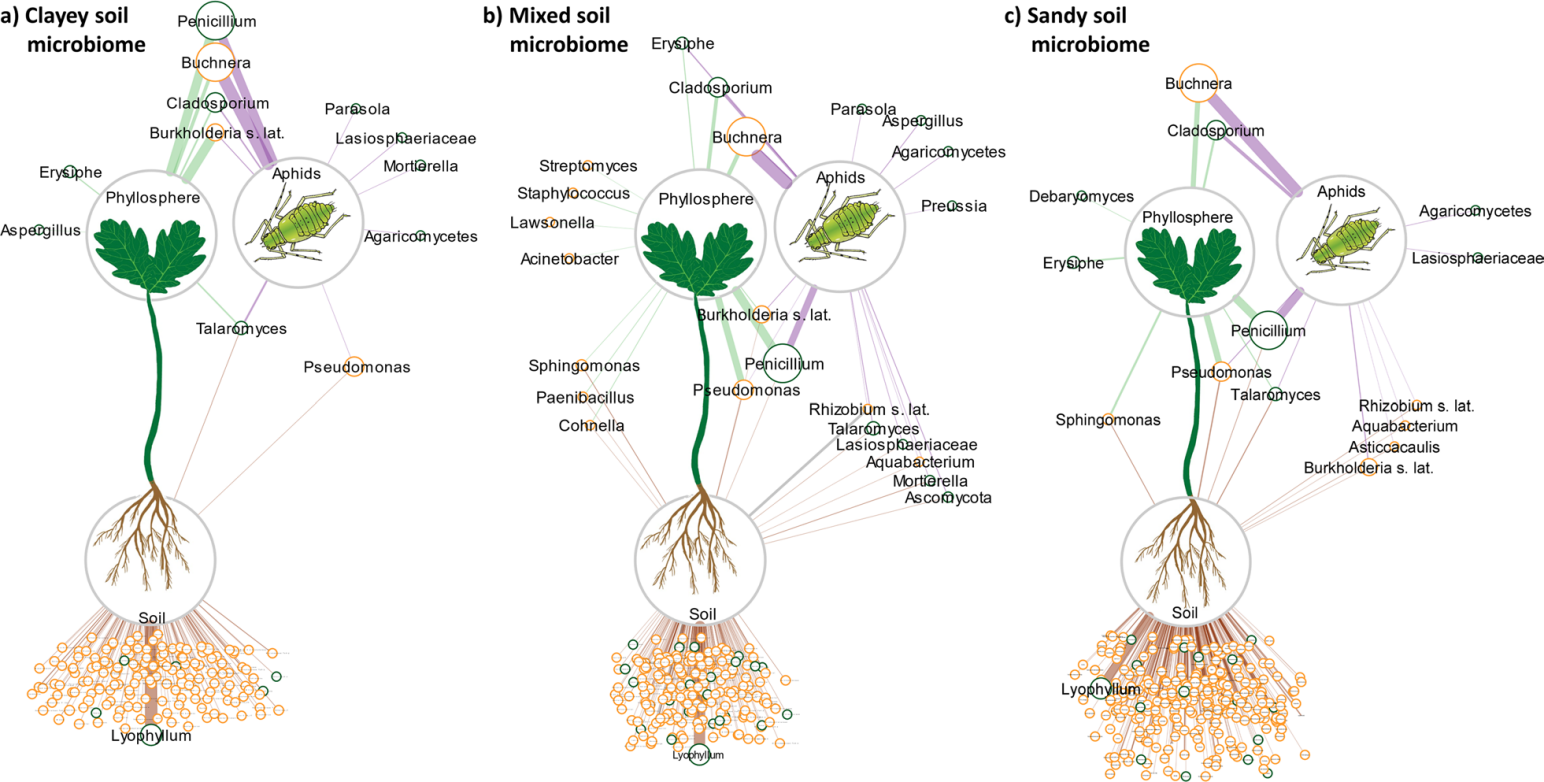
Microbial core taxa that are unique and shared by the soil, phyllosphere, and aphid compartments. Network visualization for samples originating from oak seedlings infested with aphids grown in clayey (**a**), mixed (**b**), and sandy **c** soil microbiomes. Core taxa (≥ 50% prevalence in the respective compartment-soil type combination) at genus level, with bacterial and fungal datasets combined. Edge widths corresponds to relative abundance in the respective compartment (soil, phyllosphere, or aphids). Soil taxa labels of the clayey (n = 153), mixed (n = 186), and sandy soil (n = 270) compartments were not adjusted in size to increase clarity, except for the dominant genus *Lyophyllum*

The genera *Buchnera, Penicillium* and *Cladosporium* were shared between aphids and phyllosphere, independent of soil microbiome (Fig. 5a–c). Genera partially shared by all compartments depending on soil microbiome include *Burkholderia* s. lat. (in mixed soil microbiome, Fig. 5b), *Pseudomonas* (in mixed and sandy soil microbiome, Fig. 5b, c), and *Talaromyces* (in clayey and sandy soil microbiomes, Fig. 5a, c). The plant-pathogenic fungal genus *Erysiphe* was shared in mixed soil microbiomes between phyllosphere and aphids (Fig. 5b), while it was unique to phyllosphere in clayey and sandy soil microbiomes (Fig. 5a, c). Genera that are only shared between bulk soil and phyllosphere differed between different soil microbiomes (e.g., *Sphingomonas* was only shared in mixed and sandy soil microbiomes, Fig. 5b,c), as well as genera shared between bulk soil and aphids (e.g., *Rhizobium* s. lat. = *Allorhizobium-Neorhizobium-Pararhizobium-Rhizobium*, only in sandy and mixed soil microbiomes, Fig. 5b,c).

## Discussion

In the current study, we showed that manipulating soil microbiome changed the plant phyllosphere, and subsequently the aphid microbiome. We found aphid infestation to have significant effects on phyllosphere microbiomes and soil microbiome-plant interactions, interfering with plant-mediated assembly. The implications of aphid infestation for plant-associated microbiomes depend on microbial communities in bulk soil. In this way, this study provides experimental evidence for the reciprocal influence of soil, plant, and aphid microbiomes.

We developed and tested a method for standardizing physicochemical soil properties while providing different natural soil microbiome communities in experimental setups. Soil standardization is an ongoing challenge in plant microbiome research, as is the standardization of methodological approaches like sequencing and bioinformatic pipelines [63, 64]. Microbial soil microbiota are closely intertwined with both the abiotic and biotic components of their habitat. Although the experimental use of defined, synthetic microbial communities (SynComs) and gnotobiotic plants in such settings is possible, these systems only partially mimic the complexity in microbial interactions in naturally-occurring microbiomes [65]. The hereby presented method still changes the physicochemical properties of the substrate compared to the original inoculum source, however, the natural microbial communities originating from different sources remained distinct from each other. Therefore, our method is a compromise between excluding physicochemical differences in soil as a factor while maintaining the distinctiveness of microbial soil diversity of different origins.

Soil microbiome affected plant-mediated phyllosphere microbiome assembly. Microbial assembly in plants is regarded as a non-random process governed by selective pressures within the host plant itself [9, 66], and the fact that soil microbiome shapes phyllosphere microbiomes was observed before in other plant species [7, 9, 67–69]. By investigating the effect of different microbial communities in soils of the same physicochemical properties, we could show that phyllosphere microbiome assembly is plant-mediated and thus at least partially driven by biotic factors. Still, selective pressures on microbial endophytes in oak appeared to be generally high, since microbial phyllosphere diversity was low in comparison to other tree species [70, 71] which was observed in oaks grown in microcosms [10] and under field conditions [72]. The effect of soil microbiomes on phyllosphere microbiomes was most evident for plants grown in sandy soil microbiomes. This soil was dominated by *Proteobacteria* which are known to be the most common phylum of plant microbiomes [14, 73]. Thus, experiments conducted in *Proteobacteria*-rich soils may result in more apparent effects on above-ground microbiomes. Although we found taxa dominating endophytic acorn communities as dominant taxa in the phyllosphere, namely *Pseudomonas*, *Burkholderia*, *Erwinia*, *Cladosporium* or *Penicillium* [10], we further identified bacterial biomarkers arising from soil communities in phyllospheres (e.g., *Streptomyces*, *Acinetobacter*). This experimentally confirms soil microbiomes shape oak phyllosphere microbiomes via plant-mediated assembly processes and indirectly by modulating endophytic communities even without any direct physical contact between soil and phyllosphere.

We found that the soil microbiome affected aphid microbiome assembly, even without any direct physical contact. Several studies reported the effects of soil microbiome on aphid performance before [74–76], and these effects were discussed to arise from indirect effects on plant defense systems (e.g. [77]). A similar experiment in potato and potato aphids (*Macrosiphum euphorbiae* THOMAS) revealed soil diversity to affect bacterial aphid microbiomes [7], reporting a higher effect size of soil on aphid microbiomes compared to our study. Here, the soil microbiome more evidently affected fungal communities in aphids, although the soil microbiome effect on the phyllosphere was more evident for bacteria. To our knowledge, this is the first culture-independent study investigating aphid-associated fungal communities, and we found generalistic fungal taxa common to phyllosphere surfaces in aphids. Therefore, we hypothesize aphid-associated fungi arise from direct exchanges between epifoliar and epicuticular fungi. Epifoliar fungi are considered to be generalists [78], and such fungi may be enhanced by aphid honeydew deposition, increasing chances to re-associate with aphids. The prevalence of phytopathogenic fungi (e.g., powdery mildew *Erysiphe*) in aphids may also point towards a role of aphids in their distribution, spreading spores and hyphae over the host plant or transferring them to new host plants. Leaf coverage of *Erysiphe* on oak leaves is higher when it is applied together with aphids, but lower when aphids infest the leaf three weeks before the fungus, compared to single leaf infection with *Erysiphe* [79]. Interestingly, bacterial species richness is around 11-fold higher in aphids (x̄ = 180.4 ± 112.7) than in phyllosphere (x̄ = 7.3 ± 3.7). Higher microbial diversity in herbivores than in the leaves they were consuming was reported before (e.g., [80]), and we did not remove low-abundant taxa [7], therefore, we assume that these numbers may be overestimating species richness, but not necessarily represent a data relic. Due to the limited diversity in oak phyllospheres and additional selective pressures in the digestive tract of aphids, direct uptake of soil microbes via plant sap may be frequent, but appears to only have minor effects on the autochthonous aphid microbiome.

We observed that aphid herbivory decreased the microbial alpha diversity in the phyllosphere, yet abundance was not affected. As based on the observed R^2^ values, the statement that soil microbiomes have a stronger impact on microbial phyllosphere community composition than aphid herbivory [7] could be confirmed in this pathosystem. Herbivore-associated microbiomes are known to interfere with plant metabolism and plant defense responses, thus acting as a hidden driving force of plant–herbivore coevolution [81]. While grazing insects and necrotrophic pathogens activate jasmonate (JA)- and ethylene- (ET) dependent plant defense responses, biotrophic pathogens activate salicylate- (SA) dependent pathways. Aphid herbivory however usually leads to an activation of SA-dependent plant response while JA-dependent plant response is downregulated [82, 83]. External application of JA to plants negatively affect aphid fitness [82, 83]. However, the interplay between JA-, SA-, and ET-mediated plant defense responses also affects the assembly of soil microbes [31, 84]. While JA application was observed to increase phyllosphere microbial richness and abundance, SA-mediated defenses decreased phyllosphere endophytic diversity [85, 86], consistent with our results for endophytic fungi. There are indications for aphid-associated microbes to play a role in plant defense activation, because *Buchnera*-derived proteins in aphid saliva activate plant immune responses [30]. Manipulating plant defense responses by using microbes as plant immunological ‘decoy’ was also observed in Colorado potato beetles (*Leptinotarsa decemlineata* SAY): microbes originating from their saliva triggered antimicrobial (SA-regulated) rather than antiherbivore (JA and ethylene-regulated) plant defense responses [87]. An increase in SA and a decrease in JA response-related gene expression were also observed and discussed for bacteria in aphid honeydew [88]. SA-mediated plant defense activation by aphids may also explain why powdery mildew (*Erysiphe*) leaf coverage is lower compared to control plants when aphids feed on oak seedlings beforehand [79]. Interestingly, we found an example of a potentially “misled” plant immune response in phyllosphere grown in mixed soil microbiomes. The abundance of the entomopathogenic fungus *Metarhizium brunneum* (METSCHN.) SOROKIN was only found in mixed soil microbiomes and corresponding phyllospheres. *Metarhizium* was reduced in aphid-infested phyllospheres, despite being potentially beneficial to the plant, since it can infect and kill aphids if directly applied [89]. These results indicate an untargeted, general decrease of several fungal taxa upon aphid herbivory, either arising directly via plant stress responses or indirectly by affecting soil microbiome assembly processes in the plant. The deposition of honeydew may further favor microbial generalists, masking the loss in abundance. Therefore, we hypothesize phyllosphere microbiome shifts upon aphid herbivory to be the consequence of an untargeted antifungal plant defense response, as well as honeydew deposition by the aphid.

SA-mediated plant defense responses were reported to be specifically important for rhizosphere assembly [84]. Here, we found bulk soil microbial community composition to determine whether bulk soil microbiome shifts upon above-ground herbivory. This explains, why effects of sap-sucking insects on soil and rhizosphere microbiomes were reported before [33, 90, 91], while some studies observed no effect [35]. Similar to our results, soil microbiome-dependent responses upon aphid infestation were reported in a wild tomato (*Solanum pimpinellifolium* MILL. ex DUNAL) -potato aphid (*Macrosiphum euphorbiae* THOMAS) pathosystem [92]. In contrast to our results, *Bacillaceae* were discussed as positive responders to aphid herbivory in soil. In our experiment, *Bacillaceae* responded negatively, while *Xanthobacteraceae* and *Rhizobiaceae* (ad *Proteobacteria*) responded positively in bulk soil upon aphid herbivory. Soils used by French and colleagues (2021) did not significantly differ in the relative abundance of *Proteobacteria*, therefore the exact cause for the responsiveness of soil microbiomes to herbivory remains a matter of debate. However, responses are most likely driven by plant root exudation. Oak root exudate composition is known to shift towards a higher concentration of secondary metabolites under abiotic stress [93]. Given that exudate composition changes accordingly under biotic stress, soil microbiome responses upon herbivory may be indirectly driven by the susceptibility of soil microbes towards such root exudates, or their ‘attractiveness’ for soil microbes. For the observed effects in sandy soil microbiomes, the”cry-for-help” hypothesis [94] cannot be excluded, but observations can be also explained with decreased amounts of metabolizable root exudates [95]. Firstly, taxa increased in aphid-infested sandy bulk soil are not known for antiherbivore or plant growth-promoting effects. Secondly, genera known for nitrogen fixation (*Rhizobium*, *Mesorhizobium*) are—yet not significantly—increased, indicating a more nutrient-depleted environment compared to control plants. Thirdly, bacterial species richness is higher in aphid-infested sandy bulk soil, indicating a lower selection pressure of plant root exudates to soil microbes. This however could also be interpreted as a “cry-for-help” effect, with soil microbes in sandy bulk soil being more responsive to the exuded metabolites than in the other tested bulk soil communities. Fourthly, bacterial abundance is generally lower in bulk soil of infested plants. Lastly, when combining all bulk soil data, we do not find significantly responding biomarker taxa in differential abundance analyses. Altogether, this indicates a weaker selective force of root exudates on microbes in the root periphery of aphid-infested plants compared to control plants.

## Conclusion

Soil, plant, and aphid microbiomes are in a dynamic tripartite interaction, in which the strength of effects depends on the represented microbial communities and not the physiochemical properties of soil. While directly shaping the phyllosphere and aphid microbiome using soil is possible to some extent, the effect size of the soil microbiome gradually decreases from phyllosphere to aphids. Still, soil microbes being transmitted to aphids via the plant are of interest for the biocontrol of pests, since soil or seed treatments are easier to handle, and have a less mechanical impact on agricultural plants than spray applications [96]. Herbivory has implications for phyllosphere and partially soil microbiomes, although the specific responses depend on yet unidentified soil microbiome specifics. To fully disentangle the role of soil microbiome from soil physicochemical properties in tritrophic systems, future studies could investigate the response of plant microbiomes to synthetic or otherwise defined microbial soil communities under different physicochemical soil conditions and stressors. 

## Supplementary Information

**Additional file 1**. **Supplementary Figures. Fig. S1:** Rarefaction curves for amplicon samples of each compartment. **Fig. S2:** Soil microbiome response upon aphid infestation. **Fig. S3:** Differences in microbial soil alpha diversity and abundance arising in corresponding inocula and soils (all soil treatments merged). **Fig. S4:** Overview of microbial alpha diversity and abundance in inoculum and soil treatments. **Fig. S5:** Microbial community composition development in soils depending on aphid herbivory. **Supplementary Tables. Table S1:** Source and proportion of soil mixes. **Table S2:** Pairwise PERMANOVA results for soil microbiome dependency of microbial phyllosphere community composition. **Table S3:** Pairwise PERMANOVA results for soil microbiome dependency of aphid microbiome community composition. **Table S4:** Differential abundance analysis results of soil-dependent biomarkers in aphid bacteria. **Table S5:** Differential abundance analysis results of soil-dependent biomarkers in aphid fungi. **Table S6:** Differential abundance analysis results of aphid herbivory-dependent fungal biomarkers in phyllosphere microbiome. **Table S7:** Differential abundance analysis results for aphid herbivory-dependent bacterial biomarkers in sandy soil microbiome. **Table S8:** Differential abundance analysis results for soil taxa affected by soil microbiome development from inoculum to soil and aphid herbivory. **Supplementary Methods. Methods S1:** Modified protocol of the Standard DNeasy® Blood&Tissue procedure for insects. **Methods S2:** PCR mixes and PCR conditions for amplicon and RT-qPCR. **Supplementary Notes. Notes S1:** Detailed description of dominant and biomarker taxa in soil microbial communities. **Notes S2:** Detailed description of dominant and biomarker taxa in aphid microbial communities.

## Data Availability

The dataset supporting the conclusions of this article is available in the European Nucleotide Archive (ENA) repository, accession number PRJEB50358. The code used in this study will be made available on zenodo upon acceptance of the manuscript.
