# Supplementary material for: Reciprocal influence of soil, phyllosphere, and aphid microbiomes
Source: Environ Microbiome. 2023 Jul 21;18:63. doi: 10.1186/s40793-023-00515-8 (PMC10362670; doi:10.1186/s40793-023-00515-8)
Supplement: Supplementary file 1 — Additional file 1. Supplementary Figures. Fig. S1: Rarefaction curves for amplicon samples of each compartment. Fig. S2: Soil microbiome response upon aphid infestation. Fig. S3: Differences in microbial soil alpha diversity and abundance arising in corresponding inocula and soils (all soil treatments merged). Fig. S4: Overview of microbial alpha diversity and abundance in inoculum and soil treatments. Fig. S5: Microbial community composition development in soils depending on aphid herbivory. Supplementary Tables. Table S1: Source and proportion of soil mixes. Table S2: Pairwise PERMANOVA results for soil microbiome dependency of microbial phyllosphere community composition. Table S3: Pairwise PERMANOVA results for soil microbiome dependency of aphid microbiome community composition. Table S4: Differential abundance analysis results of soil-dependent biomarkers in aphid bacteria. Table S5: Differential abundance analysis results of soil-dependent biomarkers in aphid fungi. Table S6: Differential abundance analysis results of aphid herbivory-dependent fungal biomarkers in phyllosphere microbiome. Table S7: Differential abundance analysis results for aphid herbivory-dependent bacterial biomarkers in sandy soil microbiome. Table S8: Differential abundance analysis results for soil taxa affected by soil microbiome development from inoculum to soil and aphid herbivory. Supplementary Methods. Methods S1: Modified protocol of the Standard DNeasy® Blood&Tissue procedure for insects. Methods S2: PCR mixes and PCR conditions for amplicon and RT-qPCR. Supplementary Notes. Notes S1: Detailed description of dominant and biomarker taxa in soil microbial communities. Notes S2: Detailed description of dominant and biomarker taxa in aphid microbial communities. [file 40793_2023_515_MOESM1_ESM.docx]

**Supporting Information:**

## Wolfgang et al., 2023: “Reciprocal influence of soil, phyllosphere and aphid microbiomes”

Authors: **Adrian Wolfgang, Ayco J. M. Tack, Gabriele Berg, Ahmed Abdelfattah**

The following document includes the following supporting information:

- **Supplementary Figures**
  - **Fig. S1:** Rarefaction curves for amplicon samples of each compartment
  - **Fig. S2:** Soil microbiome response upon aphid infestation
  - **Fig. S3:** Differences in microbial soil alpha diversity and abundance arising in corresponding inocula and soils (all soil treatments merged).
  - **Fig. S4:** Overview of microbial alpha diversity and abundance in inoculum and soil treatments
  - **Fig. S5:** Microbial community composition development in soils depending on aphid herbivory
- **Supplementary Tables**
  - **Table S1:** Source and proportion of soil mixes
  - **Table S2:** Pairwise PERMANOVA results for soil microbiome dependency of microbial phyllosphere community composition
  - **Table S3:** Pairwise PERMANOVA results for soil microbiome dependency of aphid microbiome community composition
  - **Table S4:** Differential abundance analysis results of soil-dependent biomarkers in aphid bacteria
  - **Table S5:** Differential abundance analysis results of soil-dependent biomarkers in aphid fungi
  - **Table S6:** Differential abundance analysis results of aphid herbivory-dependent fungal biomarkers in phyllosphere microbiome.
  - **Table S7:** Differential abundance analysis results for aphid herbivory-dependent bacterial biomarkers in sandy soil microbiome
  - **Table S8:** Differential abundance analysis results for soil taxa affected by soil microbiome development from inoculum to soil and aphid herbivory
- **Supplementary Methods:**
  - **Methods S1:** Modified protocol of the Standard DNeasy® Blood&Tissue procedure for insects
  - **Methods S2:** PCR mixes and PCR conditions for amplicon and RT-qPCR
- **Supplementary Notes:**
  - **Notes S1:** Detailed description of dominant and biomarker taxa in soil microbial communities
  - **Notes S2:** Detailed description of dominant and biomarker taxa in aphid microbial communities

## Supplementary Figures:


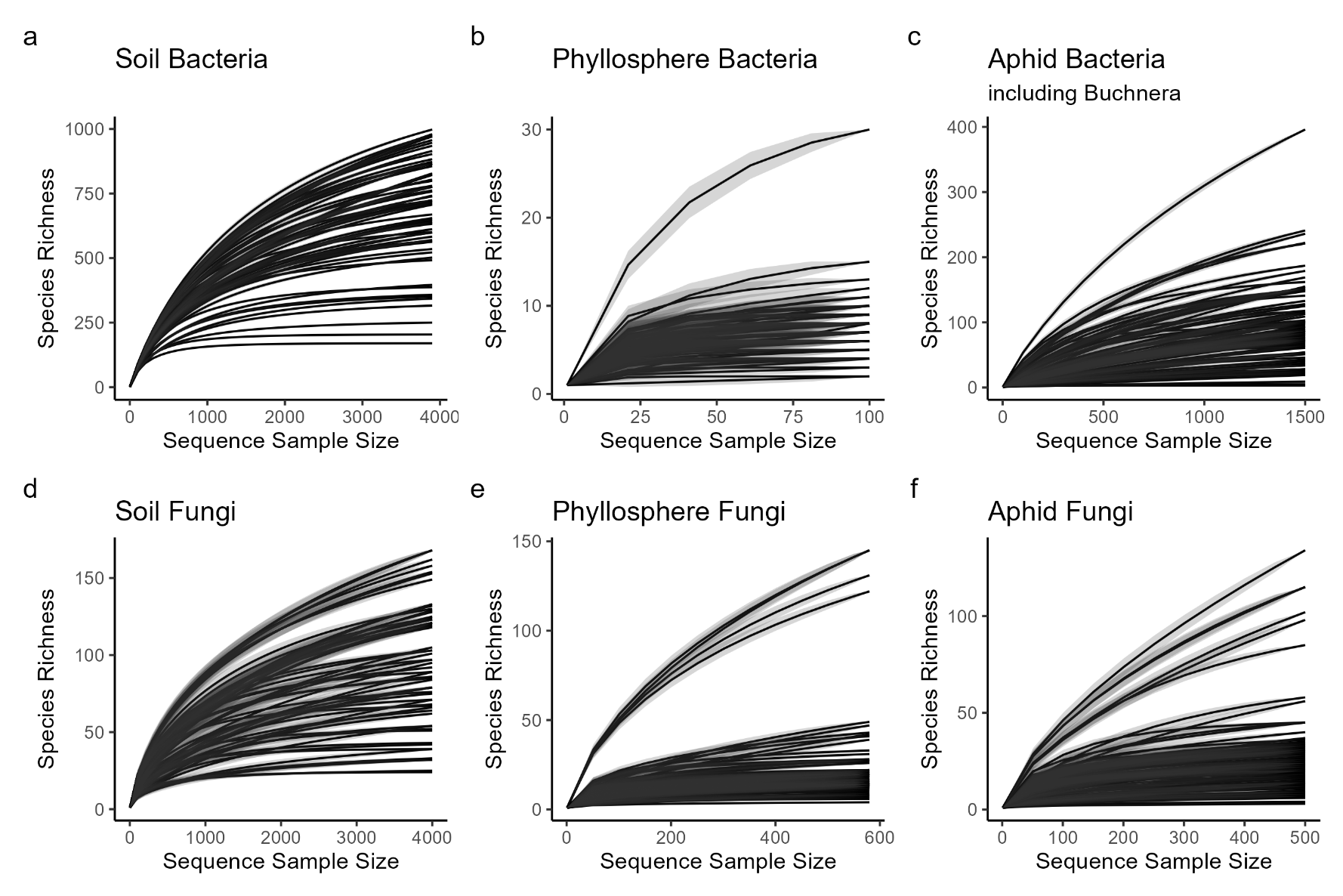


**Fig. S1:** Rarefaction curves for bacterial (a-c) and fungal (d-f) amplicon samples of each tested compartment. X-axis cutoff at correspondingly used rarefaction depth.

**
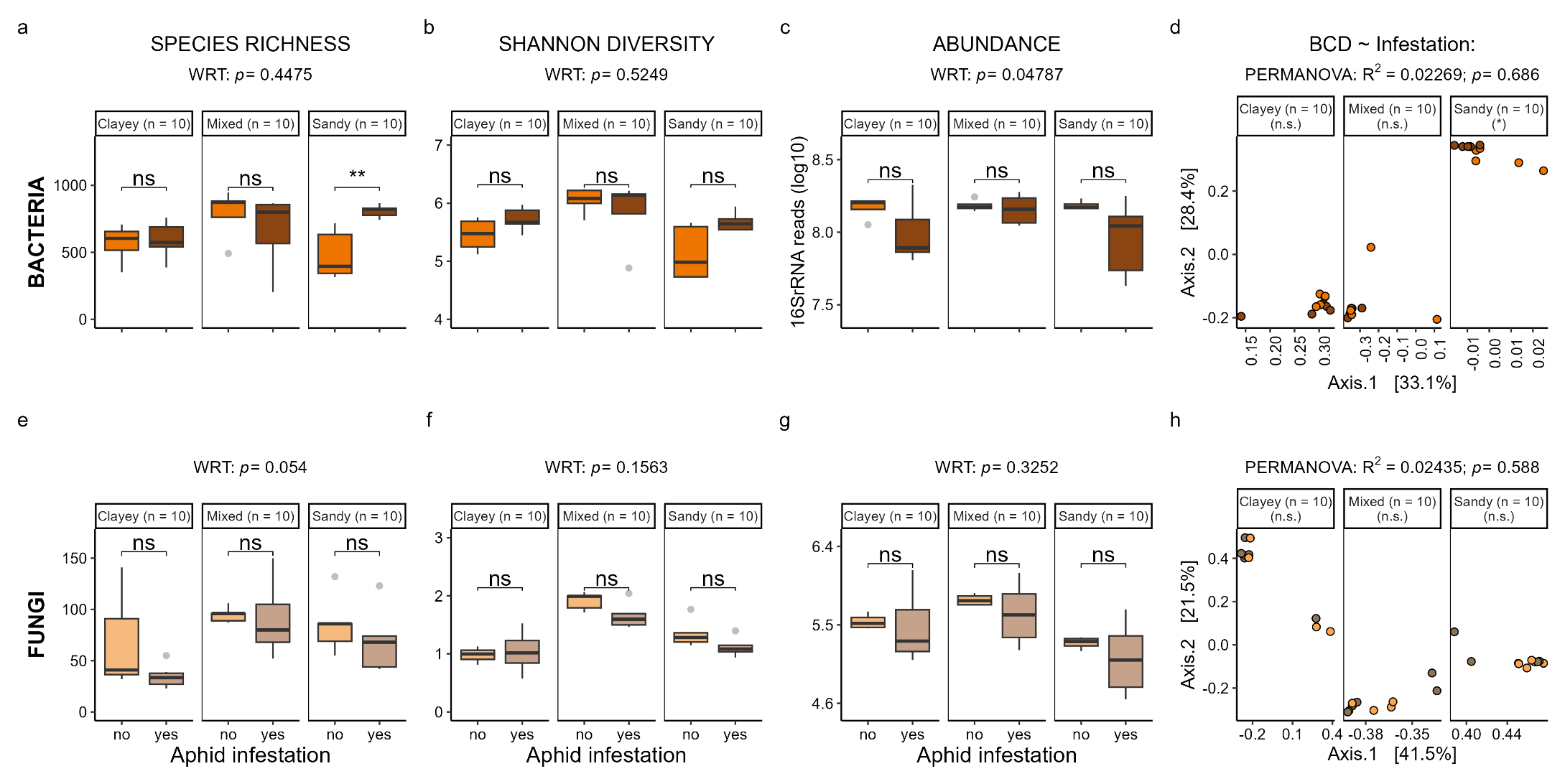
**

**Fig. S2:** Soil microbiome response upon aphid infestation in bacterial (**a-d**) and fungal (**e-h**) species richness (**a,e**), Shannon diversity (**b,f**), abundance (**c,g**) and community composition (**d,h**), faceted according to soil type; orange: control group; brown: aphid-infested. Number of samples tested (n) displayed in corresponding subplot. P-value for Wilcoxon signed-rank test for all soil types combined displayed above each plot. WRT: Wilcoxon signed-rank test; ns: not significant

**
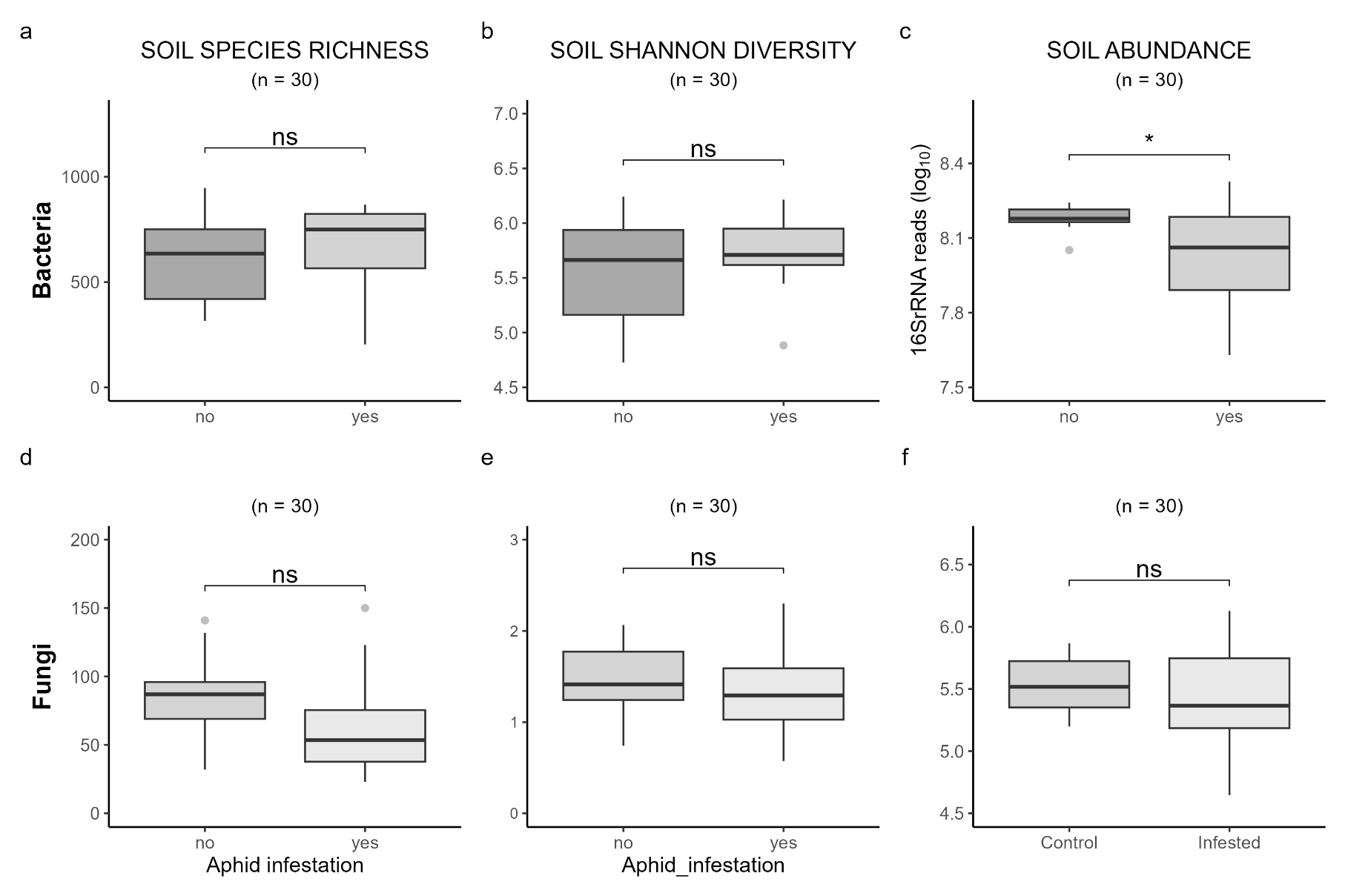
Fig. S3:** Differences in microbial soil alpha diversity and abundance arising in corresponding inocula and soils (all soil treatments merged). Effect of aphid herbivory on bacterial (**a-c**) and fungal (**d-f**) species richness (**a,e**), Shannon diversity (**b,f**), evenness (**c,g**), and abundance (**d,h**); darkgrey: control; lightgrey: soil from aphid-infested plants. Significance tested based on Wilcoxon signed-rank test. Number of tested samples indicated above each plot.

**
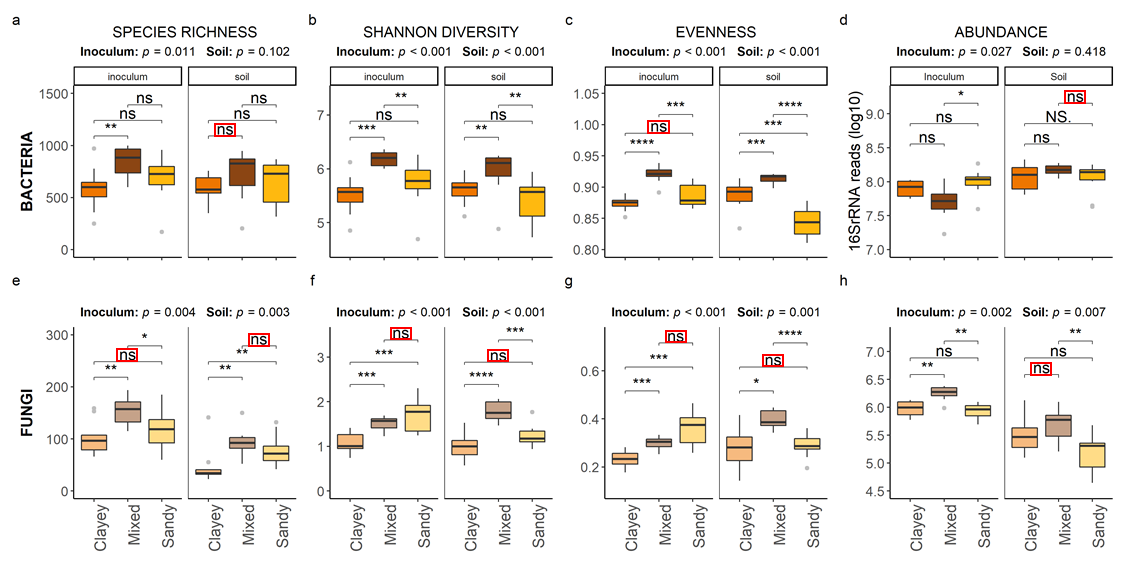
Fig. S4**: Overview of microbial alpha diversity and abundance in inoculum and soil treatments. Bacterial (**a-d**) and fungal (**e-h**) species richness (**a,e**), Shannon diversity (**b,f**), evenness (**c,g**), and abundance (**d,h**) in inoculum and soil after the experiment for all soil types separately. Differences between corresponding inocula and soils arising in the course of the experiment highlighted with red rectangles. Orange: clayey soil inoculum; dark brown: mixed soil inoculum; yellow: sandy soil inoculum.

**
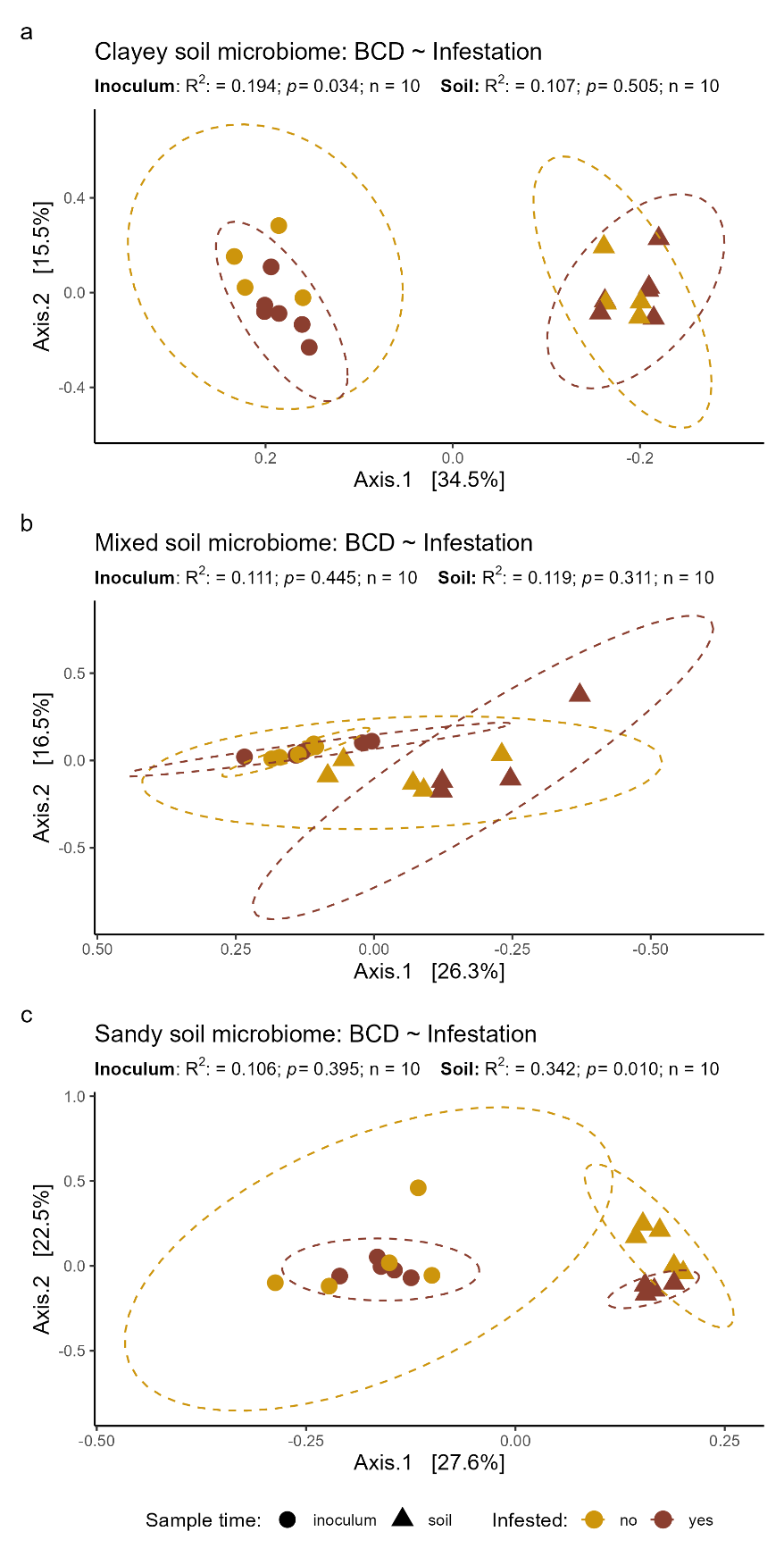
Fig. S5:** Microbial community composition development in soils depending on aphid herbivory. PCoA ordination plots based on Bray-Curtis dissimilarity (BCD) index. Communities in soils with clayey (**a**), mixed (**b**) and sandy (**c**) soil inoculum displayed separately. PERMANOVA analyses for the factor ‘aphid infestation’ performed separately for inoculum (circles) and soil communities (triangles), results are displayed on top of the plots. Inoculum and soil cluster separately in all soils alongside axis 1. Coincidental initial differences in clayey inoculum community composition (**a**) are not observable in soil communities at the end of the experiment. Only sandy soil communities (**c**) significantly differ between infested (brown) and control plants (ochre) alongside axis 2; coincidental initial differences in sandy inoculum were not observed.

## Supplementary Tables:

**Table S1**: Source and proportion of soil mixes

| **Soil type** | **Coordinates** | **Source** | **Treatment "Clayey"** | **Treatment "Mixed"** | **Treatment "Sandy"** |
| --- | --- | --- | --- | --- | --- |
| Clayey Soil | 58°56'45.1"N, 17°09'03.3"E | Tovetorp | **1.25 l, inoculum** | 1.25 l, sterilized | 1.25 l, sterilized |
| Mixed Soil | 59°21'54.0"N, 18°03'37.2"E | SU Campus | 1.25 l, sterilized | **1.25 l, inoculum** | 1.25 l, sterilized |
| Sandy Soil | 58°56'51.4"N, 17°08'48.4"E | Tovetorp | 1.25 l, sterilized | 1.25 l, sterilized | **1.25 l, inoculum** |
| Potting Soil | Commercial | Commercial | 7.5 l, sterilized | 7.5 l, sterilized | 7.5 l, sterilized |
|  |  |  | 11.25 l final soil mix | 11.25 l final soil mix | 11.25 l final soil mix |

**Table S2**: Pairwise PERMANOVA results for soil microbiome dependency of microbial phyllosphere community composition based on Bray Curtis distances. All phyllosphere communities differ significantly according to soil microbiome.

|  | **pairs** | | | **Df** | **SumsOfSqs** | **R2** | **F** | **Pr(>F)** | **sig** |
| --- | --- | --- | --- | --- | --- | --- | --- | --- | --- |
| BACTERIA | Mixed | vs | Clayey | 1 | 1.1819 | 0.25158 | 9.4122 | 0.001 | *** |
|  | Mixed | vs | Sandy | 1 | 0.5795 | 0.1188 | 4.5837 | 0.001 | *** |
|  | Clayey | vs | Sandy | 1 | 2.5665 | 0.30324 | 20.891 | 0.001 | *** |
|  |  |  |  |  |  |  |  |  |  |
| FUNGI | Mixed | vs | Clayey | 1 | 1.1853 | 0.06715 | 5.8304 | 0.001 | *** |
|  | Mixed | vs | Sandy | 1 | 1.355 | 0.08243 | 7.0973 | 0.001 | *** |
|  | Clayey | vs | Sandy | 1 | 0.5135 | 0.04237 | 3.5393 | 0.002 | ** |

**Table S3:** Pairwise adonis results for soil community-dependent aphid microbial community composition based on Bray Curtis distances. All aphid microbiomes differ significantly according to soil microbiome, except for Bacteria in aphids reared on clayey or sandy soil microbiome.

|  | **pairs** | | | **Df** | **SumsOfSqs** | **R2** | **F** | **Pr(>F)** | **sig** |  |
| --- | --- | --- | --- | --- | --- | --- | --- | --- | --- | --- |
| BACTERIA | Mixed | vs | Clayey | 1 | 0.6002 | 0.02355 | 1.5439 | 0.001 | *** |  |
|  | Mixed | vs | Sandy | 1 | 0.6447 | 0.0256 | 1.6551 | 0.001 | *** |  |
|  | Clayey | vs | Sandy | 1 | 0.4332 | 0.01775 | 1.1022 | 0.178 |  |  |
|  |  |  |  |  |  |  |  |  |  |  |
| FUNGI | Mixed | vs | Clayey | 1 | 0.5913 | 0.04201 | 2.4555 | 0.001 | *** |  |
|  | Mixed | vs | Sandy | 1 | 1.2264 | 0.07953 | 5.3566 | 0.001 | *** |  |
|  | Clayey | vs | Sandy | 1 | 0.9592 | 0.06805 | 3.9433 | 0.001 | *** |  |

**Table S4**: Differential abundance analysis results of biomarker taxa for soil microbiome in aphid bacteria. Top five taxa with the highest LDA score in the corresponding taxonomic rank. Total: Total numbers of taxa with positive abundance response in respective soil group. P-values uncorrected, except for *Micromonosporaceae* (highlighted in bold).


**Table S5:** Differential abundance analysis results of biomarker taxa for soil microbiome in aphid fungi. Top five taxa with the highest LDA score in the corresponding taxonomic rank. Total: Total numbers of taxa with positive abundance response in respective soil group). P-values uncorrected

**Table S6:** Differential abundance analysis results of fungal biomarker taxa for aphid infestation in phyllosphere. Top five taxa with the highest LDA score in the corresponding taxonomic rank. Total: Total numbers of taxa with positive abundance response in respective group). P-values corrected, taxa with uncorrected p-value removed.

|  | **Higher in uninfested Leaves (p.adj. <0.05)** | | | **Higher in infested Leaves (p.adj. <0.05)** | | |
| --- | --- | --- | --- | --- | --- | --- |
| **Rank** | **total** | **top five taxa** | **LDA score** | **total** | **top five taxa** | **LDA score** |
| Phylum | 3 | *Basidiomycota* | 5.56 | 1 |  |  |
|  |  | *Chytridiomycota* | 3.14 |  | Ascomycota | 5.73 |
|  |  | unidentified Fungi | 2.51 |  |  |  |
| Class | 11 | *Agaricomycetes* | 5.52 | 0 |  |  |
|  |  | *Leotiomycetes* | 5.5 |  |  |  |
|  |  | *Sordariomycetes* | 5.5 |  |  |  |
|  |  | *unidentified Ascomycota* | 4.96 |  |  |  |
|  |  | *Pezizomycetes* | 3.73 |  |  |  |
| Order | 29 | *Helotiales* | 5.37 | 0 |  |  |
|  |  | *Sordariales* | 5.36 |  |  |  |
|  |  | *unidentified Ascomycota* | 4.96 |  |  |  |
|  |  | *Pleosporales* | 4.94 |  |  |  |
|  |  | *Russulales* | 4.68 |  |  |  |
| Family | 51 | *unidentified Helotiales* | 5.33 | 0 |  |  |
|  |  | *Lasiosphaeriaceae* | 5.21 |  |  |  |
|  |  | *unidentified Ascomycota* | 4.96 |  |  |  |
|  |  | *Russulaceae* | 4.68 |  |  |  |
|  |  | *Sporormiaceae* | 4.62 |  |  |  |
| Genus | 72 | *unidentified Helotiales* | 5.33 | 0 |  |  |
|  |  | *unidentified Lasiosphaeriaceae* | 5.19 |  |  |  |
|  |  | *unidentified Ascomycota* | 4.96 |  |  |  |
|  |  | *Russula* | 4.68 |  |  |  |
|  |  | *Preussia* | 4.56 |  |  |  |
| Species | 76 | *unidentified Helotiales sp.* | 5.33 | 0 |  |  |
|  |  | *unidentified Lasiosphaeriaceae sp.* | 5.19 |  |  |  |
|  |  | *unidentified Ascomycota* | 4.96 |  |  |  |
|  |  | *Russula sp.* | 4.68 |  |  |  |
|  |  | *Preussia sp.* | 4.56 |  |  |  |

**Table S7:** Differential abundance analysis results of bacterial biomarker taxa for aphid infestation in sandy soil microbiome. Top five taxa with the highest LDA score in the corresponding taxonomic rank. Total: Total numbers of taxa with positive abundance response in respective group). P-values uncorrected

|  | **Higher in Sandy control Soil (p.value. <0.05, uncorrected)** | | | **Higher in sandy infested Soil (p.value. <0.05, uncorrected)** | | |
| --- | --- | --- | --- | --- | --- | --- |
| **Rank** | **total** | **top five taxa** | **LDA score** | **total** | **top five taxa** | **LDA score** |
| Phylum | 0 |  |  | 2 | BRC1 | 2.9 |
|  |  |  |  |  | WPS-2 | 2.18 |
| Class | 0 |  |  | 6 | uncultured FBP | 3.18 |
|  |  |  |  |  | *Chloroflexi* JG30-KF-CM66 | 2.97 |
|  |  |  |  |  | *Methanomicrobia* | 2.83 |
|  |  |  |  |  | *Acidobacteria* Subgroup 17 | 2.74 |
|  |  |  |  |  | *Negativicutes* | 2.55 |
| Order | 3 |  |  | 12 | *Reyranellales* | 4.09 |
|  |  | *Xanthomonadales* | 5.51 |  | *Isosphaerales* | 3.45 |
|  |  | *Opitutales* | 4.14 |  | uncultured FBP | 3.18 |
|  |  | *Propionibacteriales* | 3.59 |  | *Azospirillales* | 3.02 |
|  |  |  |  |  | *Methanosarcinales* | 2.83 |
| Family | 7 | *Rhodanobacteraceae* | 5.43 | 29 | *Xanthobacteraceae* | 4.76 |
|  |  | *Opitutaceae* | 4.14 |  | *Rhizobiaceae* | 4.69 |
|  |  | *Bacillaceae* | 3.86 |  | *Beijerinckiaceae* | 4.29 |
|  |  | *Sandaracinaceae* | 3.66 |  | *Reyranellaceae* | 4.09 |
|  |  | *Nocardioidaceae* | 3.59 |  | *Solirubrobacterales* 67-14 | 3.58 |
| Genus | 13 | *Rhodanobacter* | 5.44 | 52 | *Allorhizobium-Neorhizobium-Pararhizobium-Rhizobium* | 4.52 |
|  |  | uncultured *Sphingomonadaceae* | 4.24 |  | unidentified *Xanthobacteraceae* | 4.31 |
|  |  | *Ramlibacter* | 4.24 |  | *Duganella* | 4.21 |
|  |  | *Lacunisphaera* | 4 |  | uncultured *Xanthobacteraceae* | 4.15 |
|  |  | *Luteimonas* | 3.95 |  | *Mesorhizobium* | 4.12 |

**Table S8:** Soil taxa enriched during the experiment from inoculum soil to the soil at the end of the experiment across all soils (only taxa shown with significant Bonferroni-corrected p-value) and soil taxa affected by aphid infestation (no taxa significant after p-value correction). If taxa affected by soil development are affected by aphid herbivory, it is only observed in one of the three tested soil microbiomes.

| **Taxa increased from inoculum to soil** |  | **Taxa affected by aphid herbivory in:** | | |  |  |
| --- | --- | --- | --- | --- | --- | --- |
|  | **after p.value correction** | **Clayey Soil** | **Mixed Soil** | **Sandy Soil** | **higher in** | **after p. value correction** |
| *Arenimonas* | significant | no | yes | no | Aphid-infested | not significant |
| *Sphingomonas* | significant | no | no | no | NA | NA |
| *Ferruginibacter* | significant | no | yes | no | Aphid-infested | not significant |
| *Terrimonas* | significant | no | yes | no | Aphid-infested | not significant |
| *Sphingopyxis* | significant | no | no | no | NA | NA |
| *Flavobacterium* | significant | no | no | no | NA | NA |
| *Sphingomonadaceae_gen* | significant | no | no | yes | control | not significant |
| *Devosia* | significant | no | no | yes | Aphid-infested | not significant |
| *Gemmatimonas* | significant | no | no | no | NA | NA |
| *Brevundimonas* | significant | no | no | no | NA | NA |
| *Micropepsaceae_gen* | significant | no | no | no | NA | NA |
| *Lysobacter* | significant | no | no | no | NA | NA |

## Supplementary Methods:

**Methods S1:** Modified protocol of the Standard DNeasy® Blood&Tissue procedure for insects. Original protocol available at: <https://www.qiagen.com/us/resources/resourcedetail?id=cabd47a4-cb5a-4327-b10d-d90b8542421e&lang=en>

1. **Cell disruption**: 180µl of buffer ATL were added in a sterile ribolyzer tube with 2 big (2-3mm) and 1 small (1mm) glass beads. Aphid(s) were added to the tube and ribolyzed two times with 5.5m/s for 30 seconds using Ribolyser FastPrep-24 (MP Biomedicals, Santa Ana, California, USA). The tubes were then centrifuged at 17,250 x g (full speed) for five minute and 6°C to concentrate the foam back to a liquid state.
2. **Protein lysis**: The 180µl of liquid was vortexed, transferred to a new tube and 20 µl of proteinase K was added. Tubes were vortexed and incubated for 1h at 56 °C and 300 rpm.
3. **Denaturing enzymes**: Tubes were vortexed for 15s, 200 µl of buffer AL and 200µl pure ethanol (97-100%) was added until no white flakes were visible.
4. **Washing DNA**: Liquid was transfered to a QIAamp Mini Spin Column and centrifuged at 10,250 x g for 1 min. Flow-through was discarded.
5. Column was placed in a new 2 ml collection tube, 500 µl Buffer AW1 was added and centrifuged at 10,250 x g for 1 min. Flow-through was discarded.
6. Column was placed in a new 2 ml collection tube, 500 µl Buffer AW2 was added and centrifuged at 17,250 x g (full speed) for 5 min. Flow-through was discarded.
7. **Elution**: Column was placed in a new 1.5 ml Eppendorf tube, membrane was incubated with 40µl buffer AE for 1 min at room temperature. Tube was centrifuged at 10,250 x g and stored at -20°C until further processing.

**Methods S2**: PCR mixes and PCR conditions for amplicon and RT-qPCR

**Bacteria in soils and leaves**

**Amplicon PCR mix bacteria in soil and leaves**

30µl reaction, per sample:

- 19.15 µl PCR-grade water
- 6 µl 5 x Taq&Go Mastermix (MP Biomedicals, Illkirch, France)
- 1.2 µl of each primer [5µM]
- 0.45 µl PNA mix [1:1 pPNA and mPNA, 50µM], preheated (5min at 55°C)
- 2 µl template

**Amplicon PCR thermal cycling conditions**

- 96°C for 5 min
- 30 cycles of
  - 96°C for 1 min
  - 78°C fo 5s
  - 54°C for 1 min
  - 74°C for 1 min
- 74°C for 10 min

**RT-qPCR mix for bacteria in soil and leaf samples**

10µl reaction, per sample:

- 2.7 µl PCR grade water

- 5 µl KAPA SYBR Green 2X MM (KAPA Biosystems, Cape Town, South Africa)

- 0.3 µl PNA mix [1:1 pPNA and mPNA, 50µM], preheated (5min at 55°C)

- 0.5µl of each primer 515f/806r [10µM]

- 1µl template, diluted 1:10

**RT-qPCR thermal cycling conditions**

- 95 °C for 5 min

- 40 cycles of

o 95 °C for 20 s

o 78 °C for 5s

o 54 °C for 15 s

o 72 °C for 30 s

- Final melt curve: 72°C-95°C

**Bacteria in aphids**

**Amplicon PCR Mix for bacteria in aphid samples**

30µl reaction, per sample:

- 16µl PCR-grade water

- 6µl 5x Phusion HF buffer (New England Biolabs, Frankfurt, Germany)

- 0.3 µl dNTPs [10mM]

- 1.2µl of each primer [5µM]

- 0.3µl Phusion polymerase (New England Biolabs, Frankfurt, Germany; 2 U/µL)

- 5µl template

**Amplicon PCR thermal cycling conditions**

- 98°C for 3 min

- 35 cycles of

o 98°C for 10s

o 54°C for 20s

o 72°C for 10s

- 72°C for 10 min

**RT-qPCR mix for bacteria in aphid samples**

10µl reaction, per sample:

- 3 µl PCR grade water

- 5 µl KAPA SYBR Green 2X MM (KAPA Biosystems, Cape Town, South Africa)

- 0.5µl of each primer 515f/806r [10µM]

- 1µl template

**RT-qPCR thermal cycling conditions**

- 95 °C for 5 min

- 40 cycles of

o 95 °C for 20 s

o 54 °C for 15 s

o 72 °C for 30 s

- Final melt curve: 72°C-95°C

**Fungi in soils and leaves**

**Amplicon PCR Mix for fungi in soil and leaf samples**

30µl reaction, per sample:

- 19.6 µl PCR-grade water

- 6 µl 5 x Taq&Go Mastermix (MP Biomedicals, Illkirch, France)

- 1.8µl MgCl2 [50mM]

- 1.2µl of each primer [5µM]

- 2µl template

**Amplicon PCR thermal cycling conditions**

- 96°C for 5 min

- 30 cycles of

o 96°C for 1 min

o 58°C for 1 min

o 74°C for 1 min

- 74°C for 10 min

**RT-qPCR mix for fungi in soil and leaf samples**

10µl reaction, per sample:

- 3 µl PCR grade water

- 5 µl KAPA SYBR Green 2X MM (KAPA Biosystems, Cape Town, South Africa)

- 0.5µl of each primer ITS1f/ITS2r [10µM]

- 1µl template

**RT-qPCR thermal cycling conditions**

- 95 °C for 3 min

- 40 cycles of

o 95 °C for 5 s

o 58 °C for 35 s

o 72 °C for 5 s

- 72°C for 10 min

- Final melt curve: 72°C-96°C

**Fungi in aphids**

**Amplicon PCR Mix**

30µl reaction, per sample:

- 14.2µl PCR-grade water

- 6µl 5x Phusion HF buffer (New England Biolabs, Frankfurt, Germany)

- 0.3µl dNTPs [10mM]

- 1.2µl of each primer [5µM]

- 1.8µl MgCl2 [50mM]

- 0.3µl Phusion polymerase (New England Biolabs, Frankfurt, Germany; 2 U/µL)

- 5µl template

**Amplicon PCR thermal cycling conditions**

- 98°C for 3 min

- 35 cycles of

o 98°C for 10s

o 58°C for 20s

o 72°C for 10s

- 72°C for 10 min

**RT-qPCR mix for aphid samples**

- 10µl reaction, per sample:

- 3 µl PCR grade water

- 5 µl KAPA SYBR Green 2X MM (KAPA Biosystems, Cape Town, South Africa)

- 0.5µl of each primer ITS1f/ITS2r [10µM]

- 1µl template

**RT-qPCR thermal cycling conditions**

- 95°C for 3 min

o 40 cycles of

o 98°C for 5s

o 58°C for 35s

o 72°C for 5s

- 72°C for 10 min

- Final melt curve: 72°C-96°C

## Supplementary Notes:

**Notes S1:** Detailed description of soil community composition

Archaeal reads in inoculum soil before the experiment ranged from 0.15 – 1.6% r.a. (relative abundance). Bacterial community was dominated by *Proteobacteria* (32-46 % r.a., *Phenylobacterium*, *Sphingomonas* and *Ramlibacter* highly abundant), *Firmicutes* (9-31% r.a., *Brevibacillus*, *Tumebacillus* and *Paenibacillus* highly abundant), *Actinobacteria* (6-14% r.a., *Conexibacter* highly abundant), *Bacteroidetes* (4-11% r.a., *Pedobacter* abundant), and *Verrucomicrobia* (3-10% r.a., *Chthoniobacter* high abundant). Fungal primers did also amplify reads of *Rhizaria* (unidentified *Cercozoa*, 0-6.6%, r.a.) which were kept in the dataset. Fungal community in both soil groups was dominated by the ectomycorrhizal genus *Lyophyllum* (ad *Basidiomycota*, 43-96% r.a.) and *Mortierella* (ad *Mortierellamycota*, 0-19 % r.a.).

Mixed soil displayed the highest richness, Shannon diversity and evenness in both bacterial and fungal communities across the three soil types. Abundance (log10-transformed qPCR reads) did not differ significantly between soils in bacteria, but in fungi abundance was higher in mixed soil. Differential abundance analyses identified 207, 333, and 198 bacterial biomarkers of all taxonomic ranks and 46, 227, and 93 fungal biomarkers for clayey, mixed and sandy soil (without inoculum), respectively. Clayey soil showed significantly higher relative abundance of *Gemmatimonadetes*; mixed soil had significantly higher relative abundance in *Archaea*, *Acidobacteria* and *Actinobacteria*; sandy soil had significantly higher relative abundance in *Alpha*- and *Gammaproteobacteria*. In general, we observe a treatment-dependent community in the three soil types.

Relative abundance of *Proteobacteria* (32-46 vs. 45-76% r.a.) and *Bacteroidetes* (4-11 vs. 9-21% r.a.) generally increased in soil, while *Firmicutes* (9-31 vs. 1-14% r.a.), *Actinobacteria* (6-14 vs. 1-10% r.a.), and *Verrucomicrobia* (3-10 vs. 2-8% r.a.) decreased in the course of the experiment. In fungi, especially the genus *Rasamsonia* (*R. aegroticola* and *R. pulvericola*, ad *Ascomycota*) was only highly (>4% r.a.) abundant in inoculum soil. Differential abundance analysis identified 434 bacterial (176 higher in soil, 258 higher in inoculum) and 131 (123 higher in inoculum, 8 higher in soil) fungal biomarkers significantly differing between the two time points. When comparing tested alpha diversity metrics across all soils, bacterial abundance significantly increases, while fungal community display a significant decrease in richness and abundance, and evenness increases. All soil types at both time points (before/after the experiment) differed significantly according to pairwise PERMANOVA results. Both soil type and time point significantly explain about 55-60% of the variation within both bacterial and fungal Bray Curtis soil dissimilarity matrices. In general, we observed a soil type-dependent community development in the course of the experiment, with mainly an increase in bacterial abundance and fungal evenness, and a decrease in fungal abundance being consistent across all soil types.

**Notes S2**: Description of aphid community composition

Bacterial aphid-associated communities were dominated by *Buchnera* (1.5- 99% r.a.). Apart from the primary endosymbiont *Buchnera*, *Burkholderia* s. lat. (0-74% r.a.; nine samples > 10%), and *Pseudomonas* (<0.1-98% r.a., three samples > 10%) accounted partially for very high abundances. Interestingly, archaeal reads assigned to the family *Halomicrobiaceae* were found in all except five aphid samples. Fungal aphid-associated communities were dominated mainly by opportunistic air-borne taxa, that were partially high abundant in few samples and as well found in control samples (*Penicillium* (<0.1-100% r.a.), *Cladosporium* (1-96% r.a.), *Talaromyces* (0-63% r.a.), *Mortierella* (0-23% r.a.), and *Alternaria* (0-12% r.a.). Interestingly, animal- (e.g., *Simplicilium* (0-80% r.a.)) and plant-pathogenic fungi (e.g., *Erysiphe* (0-69% r.a.)) were also frequently found in relatively high abundances.
